# Muscle or Fascial System Lesion (Part II): The Medial Gastrocnemius and the “Tennis Leg” Paradigm

**DOI:** 10.7759/cureus.93994

**Published:** 2025-10-07

**Authors:** Saverio Colonna, Fabio Casacci, Paolo Minafra, Nicola Basile

**Affiliations:** 1 Rehabilitation Medicine, Spine Center, Bologna, ITA; 2 Research and Development, Osteopathic Spine Center Education, Bologna, ITA; 3 Radiology, Affidea Modena, Bologna, ITA; 4 Physiotherapy, Spine Center, Bologna, ITA

**Keywords:** achilles tendon, medial gastrocnemius strain, muscle strain injury, myoaponeurotic injury, myofascial junction, myotendinous junction, neuromuscular coordination, return-to-play, tennis leg, ultrasound evaluation

## Abstract

Muscle injuries have traditionally been interpreted as disruptions of contractile fibers. Increasing evidence, however, highlights the central role of the intramuscular connective tissue system - including endomysium, perimysium, epimysium, aponeuroses, and extracellular matrix - in force transmission, injury susceptibility, and recurrence risk.

Medial gastrocnemius strain, or “tennis leg,” exemplifies how myoconnective architecture dictates injury mechanics. The biomechanical interplay between the medial gastrocnemius (MG) and lateral gastrocnemius (LG) and between the MG and soleus (Sol) may represent key determinants of tissue failure through aponeurotic shear and displacement mismatches. Biological healing times, including the expected phases of inflammation, proliferation, and remodeling, provide a framework for safe return-to-play and help tailor rehabilitation timing according to lesion severity and location.

Ultrasound imaging is a valuable tool to precisely localize the lesion, distinguish contractile from connective tissue involvement, and guide clinical decision-making, allowing monitoring of tissue continuity, early scar formation, and readiness for progressive loading. This etiopathogenetic framework has direct therapeutic implications. Rehabilitation should include a progressive therapeutic exercise program - from isometric to concentric, eccentric, and plyometric loading - complemented by neuromuscular coordination drills. Such programs must be calibrated by joint angle and mechanical demand to improve viscoelastic properties and optimize connective tissue adaptation, thereby enhancing fascial resilience and neuromuscular efficiency.

Complementary manual therapy, aimed at restoring fascial continuity, correcting articular restrictions (particularly at the subtalar joint), and optimizing posterior myofascial chain function, represents an essential component of treatment. When integrated with ultrasound-guided monitoring and aligned with biological healing timelines, these approaches might support safer functional recovery, contribute to lowering recurrence risk, and offer a preventive framework for athletes at high risk of re-injury.

## Introduction and background

Reference to Part I concepts

In the first part of this work [1], a multilevel analysis was proposed, ranging from the histological to the clinical domain, with the aim of redefining the understanding of muscle and fascial injuries (Table 1).

**Table 1:** Key histological and clinical insights from Part I This table summarizes the principal histological and clinical insights described in Part I [1], highlighting the connective tissue structures most frequently involved in strain injuries, their biomechanical role, and the related clinical implications. These concepts provide the rationale for the fascia-inclusive perspective adopted in the present review.

Structural focus	Histological / biomechanical evidence	Clinical implications
Endomysium and perimysium	Provide the primary framework for force transmission at the micro level	Micro-disruptions explain strain injuries without complete fiber rupture
Myoaponeurotic junction	Frequent site of lesion; connective tissue continuity stressed	Common site of pain in “tennis leg” and related calf injuries
Aponeurosis	Stores elastic energy, modulates fascicle behavior under load	Overload can trigger non-uniform strain → relevant for imaging & rehab
Fascial system (epimysium & fascia)	Integrates muscles into functional chains	Guides interpretation of injury beyond muscle-only paradigm
Continuum from micro to macro	Lesions often represent detachment/strain of connective scaffolds	Supports fascia-inclusive rehabilitation and preventive strategies

It was highlighted that the so-called “muscle injury” does not necessarily correspond to a rupture of contractile fibers, but may predominantly involve the connective structures that form the supporting architecture of the muscle, particularly the endomysium, perimysium, and aponeuroses [2]. These components not only fulfill a passive mechanical role but also actively participate in the transmission and distribution of forces, both in series and in parallel, thereby contributing to the protection of muscle fibers [3].

Since muscle and connective tissue interact closely, it is hypothesized that myofascial continuity is not merely passive but also actively contributes to absorbing stretching forces [4]. This concept sets the stage for a more focused discussion on the myotendinous junction (MTJ), where the transmission of force between muscle and tendon is critical.

Butler et al. [5] provide evidence that fascia and tendon share many mechanical properties, reinforcing the idea that the MTJ represents a key site in modulating forces.

## Review

Methods

This article was conceived as a narrative review rather than a systematic review, given the wide range of perspectives considered (histology, biomechanics, pathophysiology, imaging, and rehabilitation), with particular emphasis on the ultrasonographic assessment of fascial involvement.

The literature search was updated until July 2025 and included peer-reviewed articles in English retrieved from PubMed, Scopus, and Google Scholar. Search strategies combined the following keywords: tennis leg, medial gastrocnemius strain, muscle strain injury, myoaponeurotic injury, myofascial junction, aponeurosis, ultrasound diagnosis, rehabilitation. Additional studies were identified from the reference lists of relevant articles.

Inclusion criteria were peer-reviewed articles in English or Italian addressing anatomical, pathophysiological, diagnostic, or therapeutic aspects of medial gastrocnemius injuries. Exclusion criteria were non-peer-reviewed materials and studies unrelated to the triceps surae complex.

The core evidence base of this review was limited to peer-reviewed articles. However, a limited number of classical textbooks and academic monographs were also referenced selectively for terminological clarification, historical background, and conceptual framing, rather than for primary empirical evidence

All retrieved records were imported into Zotero reference management software, where duplicates were removed. Screening of titles and abstracts was performed independently by two reviewers (S.C. and F.C.); full texts of eligible articles were then assessed for compliance with inclusion criteria and relevance to the review objectives. Discrepancies were resolved by consensus or consultation with a third reviewer (P.M.).

Debate: “fascial injury” vs. “muscle injury” at the myotendinous junction

The MTJ represents a complex transitional area where muscle fibers insert into the connective structures that transmit force to the tendon. In this region, a high concentration of a specific collagen type (type XXII), which is scarcely present in muscle and tendon, has been identified [6,7].

Traditionally, lesions at this site have been interpreted as ruptures of muscle fibers caused by the high mechanical stress generated during intense eccentric contractions [2]. However, more recent imaging studies and clinical observations suggest that, in many cases, the damage may primarily involve the fascial components of the MTJ - such as the aponeuroses and the endomysial and perimysial connections - rather than the contractile fibers themselves [8,9].

This perspective has fueled a debate in the international literature: on one side, the classical view focused on the vulnerability of muscle fibers; on the other, an emerging interpretation that acknowledges the primary role of connective tissue in the genesis of injury and in the modulation of force transmission. Understanding whether the primary damage involves muscle fibers or connective tissue is not merely a theoretical exercise: these differences have direct implications for diagnosis and rehabilitation. In light of this, it is noteworthy to observe how muscle injuries are distributed in high-intensity sports.

Epidemiology of muscle injuries in high-intensity sports

Muscle injuries represent one of the most frequent types of sports-related injuries, with an incidence ranging from 10% to 55% of all reported traumatic events [10-12]. Muscle injuries may result from contusions, strains, or, more rarely, lacerations [11,12]. Muscle lacerations are indeed the least common form, since more than 90% of sport-related muscle injuries are contusions or strains [12].

Eccentric forces exceeding 20% of the average maximal isometric force are sufficient to cause rupture at the muscle-tendon interface, which explains why this type of lesion predominantly occurs during physical activity [13]. This represents a significant challenge for sports medicine, where at least one-third of all sport-related injuries are due to muscle strains [14].

A muscle contusion occurs following a sudden and intense compressive force, such as a direct impact on the muscle, typically in contact sports. In contrast, strains are more frequently observed in activities such as sprinting or jumping [11,15]; in this case, excessive tensile force applied to the muscle produces overload of the muscle fibers and consequently a localized rupture, usually in proximity to the MTJ.

Strains mainly affect superficial biarticular muscles such as the rectus femoris, semitendinosus, and gastrocnemius [11,15,16]. In clinical practice, indirect structural muscle injuries represent the most frequent type and tend to predominantly affect specific muscle groups of the lower limbs in athletes [17]. In these muscle groups, the reuse of energy - defined in the literature as the stretch-shortening cycle (SSC) - is important for the efficiency of fundamental physical activities (walking, running, jumping, etc.). The effectiveness of this process depends on stiffness and hysteresis (the ratio between returned energy and dissipated energy). High stiffness and low hysteresis promote greater energy recovery and improve efficiency in SSC-related movements such as jumping and running [18].

In the comparison between jumps performed with and without a pre-stretching phase, the greater height commonly observed in countermovement jumps could be at least partially attributed to increased muscle activation, resulting from the rapid stretch of intrafusal fibers and the subsequent activation of α-afferents [9]. However, the importance of the stretch reflex has been questioned by other studies that did not observe significant changes in electromyographic (EMG) activity associated with active muscle stretching [19,20]. These findings have led to the hypothesis of a saturation phenomenon, according to which, under maximal contraction conditions, muscles respond only to a limited extent to transient neural stimuli [20,21]. In particular, Svantesson et al. [22], when comparing EMG activity during isokinetic plantarflexion in concentric conditions, with isometric preload, and during maximal eccentric contraction, observed unchanged or lower values when the movement was preceded by muscle action.

The latency time of the reflex response is a key factor in determining whether the increased jump height is due to a direct contribution to motoneuron activity or to an increase in passive muscle stiffness, which would enhance the system’s ability to exploit stored elastic energy [9,23]. In the study by Walshe et al. [23], the absence of significant differences in normalized EMG values across tests suggests that, for the major lower limb muscle groups, the contribution of myoelectric mechanisms to the increase in work during the SSC condition was minimal.

From the above analysis, it can be inferred that part of the tension generated during explosive movements with pre-stretching may specifically involve intra- and extra-muscular connective components.

As reported in the first part of this article [1], biomechanical studies on models and in vivo estimate that more than 30% of mechanical energy during gait is stored and passively returned by elastic structures (tendons and intramuscular connective tissue), thereby reducing the active metabolic load of muscles [24-26]. Elastic materials are those with properties similar to a spring. When subjected to loading, they accumulate strain energy through modifications of molecular bonds and conformational changes in the tertiary or quaternary structure of proteins. These structures constitute the parallel and series elastic elements of the muscle, connected to the tendon, which represents the ultimate series elastic element [27]. During the unloading phase, the stored elastic energy is released and may contribute to the mechanical movement of the body or body segment, reducing the work required by the muscle.

Focus on soccer and sports involving explosive movements

Muscle strain injuries represent one of the most frequent conditions in high-intensity sports such as soccer. Epidemiological studies [28-30] consistently report a prevalence of injuries in soccer affecting the muscles of the lower limbs, with particular involvement of the hamstrings and the triceps surae (TS).

**Figure 1:**
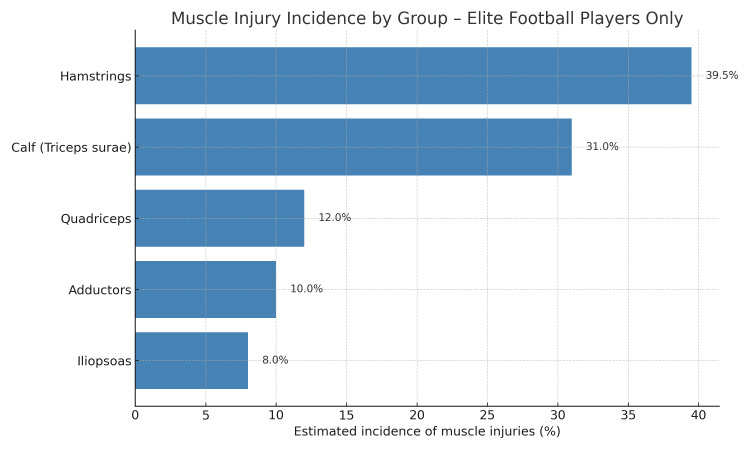
Incidence of muscle injuries in professional soccer players Data from epidemiological studies [28-30] show that nearly 70% of injuries affect the posterior chain of the lower limb.

Figure 1 shows a comparative analysis of the incidence of muscle injuries in professional soccer players, derived from epidemiological studies published on the subject [28-30], highlighting that almost 70% of injuries are localized in the posterior chain of the lower limb.

The predominance of hamstring and calf injuries in elite soccer players appears to reflect a broader trend also observed in other high-intensity sports, such as athletics [31]. In soccer players, an increase in incidence with age has been reported only for calf muscle injuries; no such trend has been observed for hamstring, quadriceps, or hip/groin strains [14]. This distribution reflects the biomechanical demands associated with sprinting, deceleration, and directional changes, which are typical of sports activities that involve explosive use of the lower limb. Although most of the literature has focused on fusiform or unipennate muscles, such as the hamstrings, the TS - a bipennate muscle with a digastric arrangement - also appears to be frequently subject to strain injuries and deserves specific biomechanical and clinical attention.

The present work aims to explore the structural, functional, and clinical characteristics of injuries involving digastric pennate muscles, with particular reference to the medial gastrocnemius (MG), a condition reported in the literature under the name tennis leg (TL).

Tennis leg

Given that the aforementioned studies highlight that the calf muscles are among the most prone to injuries in athletes, second only to the hamstrings, this section will examine this pathology in greater detail to better investigate the anatomo-histological site where the lesion occurs.

Definition

TL represents an acute injury of the calf caused by high mechanical stress. The etiology of this condition has been debated since Powell first described it in 1883 [32], and for years it was believed that TL was due to rupture of the plantaris tendon. According to Bright et al. [33], approximately two-thirds of these injuries involve the transitional zone between the medial head of the gastrocnemius muscle and the deeper soleus (Sol) muscle. Currently, the term “tennis leg” is generally used to refer to a partial or complete rupture of the connective linkage between these two muscle structures [34]. The remaining lesions are located in the lateral head of the gastrocnemius, within its muscle belly, or more rarely in the Sol.

More recent studies [35-37] therefore indicate as the main cause the rupture of the medial head of the gastrocnemius at the myotendinous junction.

Functional anatomy of the triceps surae

Gross Anatomy of the Triceps Surae

**Figure 2:**
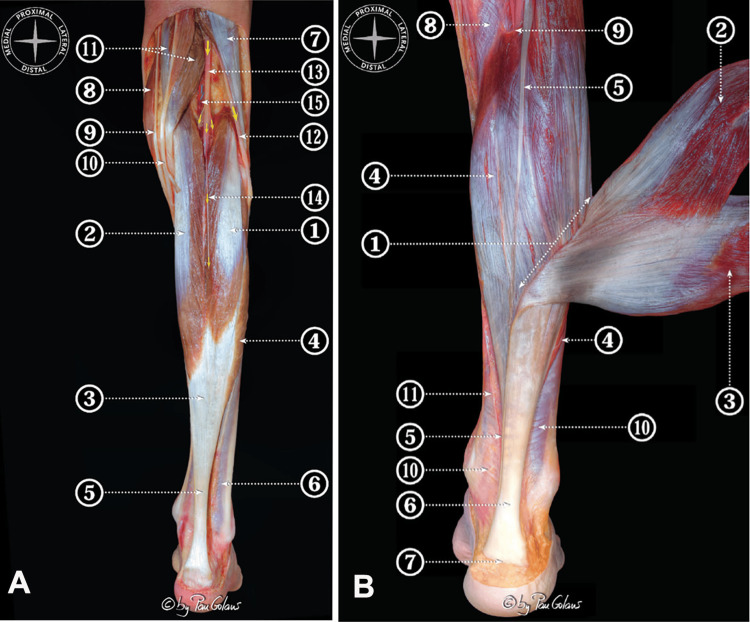
Posterior view of the triceps surae (TS) obtained from a superficial dissection of the leg and popliteal region, with adjacent anatomical structures identified A) 1, lateral head of gastrocnemius muscle; 2, medial head of gastrocnemius muscle; 3, gastrocnemius aponeurosis; 4, soleus (Sol) muscle; 5, calcaneal tendon; 6, posterior deep fascia of the leg; 7, biceps femoris muscle; 8, sartorius muscle; 9, gracilis tendon; 10, semitendinosus tendon; 11, semimembranosus muscle; 12, common peroneal nerve; 13, tibial nerve and branches; 14, sural nerve; 15, popliteal artery and vein. B) Muscular dissection of the leg to show the components of the TS (gastrocnemius muscles is rejected to reveal Sol posterior aponeurosis). 1, area of insertion of the gastrocnemius aponeurosis into the Sol posterior aponeurosis (conjoint junction); 2, lateral head of gastrocnemius muscle; 3, medial head of gastrocnemius muscle; 4, Sol posterior aponeurosis; 5, plantaris tendon; 6, calcaneal tendon; 7, insertional area of the calcaneal tendon; 8, popliteus muscle; 9, tendinous arch of the Sol muscle; 10, posterior deep fascia of the leg; 11, medial intermuscular septum. Images reproduced with permission from Dalmau-Pastor et al. [38].

The muscle-tendon structure of the human TS is anatomically and functionally complex. It consists of three distinct muscle compartments (Figure 2) that converge, through their respective aponeuroses, into a single common tendon, the Achilles tendon (AT), which inserts on the calcaneus [38].

In humans, the combined forces generated by these muscles during walking are transmitted to the AT and may reach values between 1,400 and 2,600 N, and between 3,100 and 5,330 N during running [39-41]. The AT is subjected to forces exceeding 12 times body weight during running [42] and to stretches up to 16% during single-leg jumping, in vivo [43]. It is estimated that it can store up to 35% of the total energy lost and recovered during locomotion [24,44]. A deeper understanding of the intrinsic contractile properties of the complex structure of the TS may therefore facilitate the development of new therapeutic models or targeted preventive interventions.

**Figure 3:**
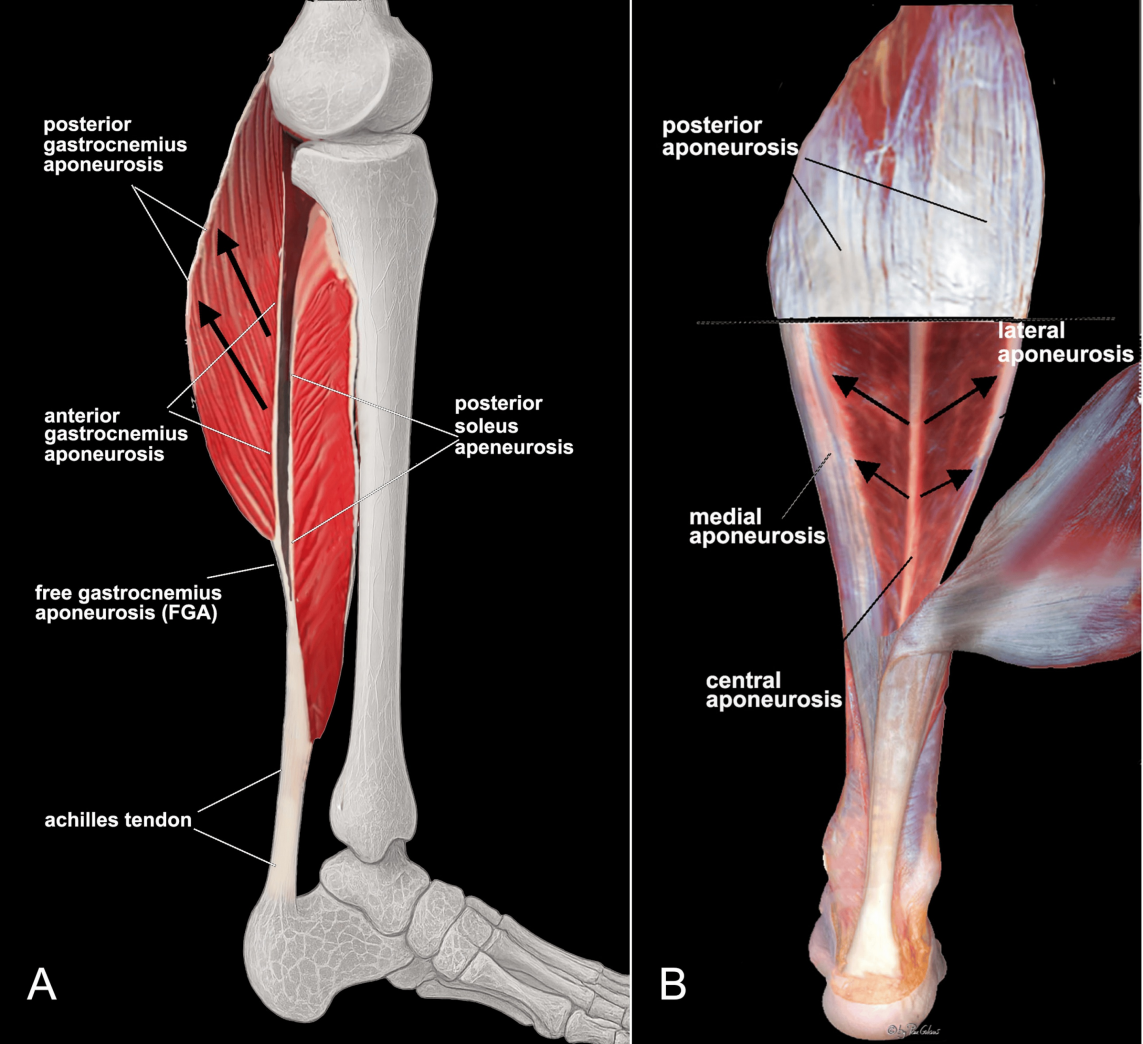
Schematic representation of the tension vectors generated by contraction of the triceps surae A) Lateral view after sagittal section of the gastrocnemius (Gm) and soleus (Sol); B) view of the Sol after removal of the digastric muscles and the superficial posterior portion. Black arrows indicate the direction of the tensile forces of the myofibers due to the pennation angle. Panel A: Image credit: Author Saverio Colonna. Original figure created by the author. Panel B: Reproduced with permission from Dalmau-Pastor et al. [38].

Since the TS includes the gastrocnemius muscles, which span both the ankle and knee joints, and the Sol muscle, which acts exclusively on the ankle, the relative contribution of each muscle to the force transmitted to the tendon varies according to the degree of knee flexion [45]. It should also be emphasized that the plane of action of the gastrocnemius and Sol is oriented on orthogonal planes. The gastrocnemius, with its posterior insertion on the proximal aponeurosis and anterior insertion on the distal aponeurosis, acts primarily in the sagittal plane (Figure 3A).

The Sol, proximally, has two insertions: the medial aponeurosis, attached to the tibia, and the lateral aponeurosis, attached to the fibula; its bipennate fibers converge distally into the central tendon, acting mainly in the coronal plane (Figure 3B).

Aponeurotic mechanics

Regarding the longitudinal direction of the aponeurosis, the portions located near the proximal and distal ends of the muscle belly are subjected to forces generated by most of the fibers, whereas the regions near its termination are stressed by a much smaller number of fibers [46]. During contraction, the aponeurosis therefore tends to lengthen more in the areas adjacent to the ends of the muscle belly and to a lesser extent at the termination. By contrast, Young’s modulus did not show significant differences among the different regions, although a tendency toward lower values was observed near the terminations of the anterior and posterior gastrocnemius aponeuroses [47]. Thus, site-dependent stiffness differences appear to be related to variable aponeurosis thickness rather than intrinsic material properties [47]. It has also been reported that the longitudinal stiffness of the aponeurosis influences fascicle behavior and likely enhances force production [48], suggesting that the aponeurosis plays an active role in modulating fascicle behavior during movement.

Muscle-tendon dynamics

An in vitro study demonstrated that when the individual muscles of the TS are activated in isolation, differences can be observed in the forces transmitted to the medial and lateral components of the Achilles tendon.

Lehr et al. [49] showed in vivo that during fixed-muscle contractions with targeted electrical stimulation, isolated activation of the Sol produces a non-uniform displacement in the Achilles tendon, significantly greater compared with MG activation (approximately +49%), supporting the idea that the TS muscles transmit force differentially to their respective subtendinous components.

In addition, non-uniform variations were observed in the estimated lengths of the different muscle compartments of the TS [49]. A heterogeneous shortening of the muscles, associated with a non-uniform distribution of tendon force, could theoretically generate intratendinous shear strain and produce sliding between tissue planes parallel to the direction of applied force.

Passive vs active conditions

At present, intratendinous shear strain induced by muscle contraction is difficult to quantify in vivo. However, it is possible to assess, using ultrasound, the displacement of the insertional aponeurosis during muscle contraction [46,50,51]. Since the collagen fibers of the aponeurosis fuse distally to form the structure of the free tendon, any differences in aponeurotic displacement may manifest as shear strain at the tendon itself.

Experimental studies [52] conducted on the MG of the rat demonstrated that the aponeurosis elongates more under passive than active conditions, reaching extensions of up to 10% when the muscle is stretched from slack length to optimal length. In comparison, the same stretch under electrical activation produces less aponeurotic elongation, despite equivalent muscle length.

During passive stretch, the aponeurosis is significantly more extensible and suitable for storing elastic energy compared with elongation induced by active muscle contraction, since muscle contraction induces a biaxial loading that increases its stiffness [48]. The results show that the extent of muscle damage is correlated with the amplitude of passive stretch during contraction [53].

This behavior suggests that, under passive conditions, the connective component exhibits greater compliance and thus a superior capacity for elastic energy storage. In other words, the potential energy stored as elastic deformation is greater during passive movement compared with active contraction, where the overall stiffness of the system limits connective tissue deformability.

These findings support the hypothesis that passive stretching phases may represent a critical moment for mechanical overload of parallel fascial structures, including the aponeurosis [54,55].

The role of passive elastic components

The importance of the passive elastic component in locomotion can be emphasized by the case of amputee runners: although up to six muscles of the lower leg are absent, running is still possible thanks to the replacement of elastic tissues with prosthetic springs. This observation suggests that passive structures may play a more crucial role than the contractile component itself [56,57].

Clinical signs and symptoms

TL is more frequently observed in middle-aged, poorly trained individuals undergoing intense physical activity [36,58]. The clinical picture includes sudden calf pain, often accompanied by the perception of a “crack” felt or palpated in the medial portion of the posterior calf, or by the sensation that someone has struck the back of the leg. Swelling appears within 24 hours, and physical examination may reveal a palpable defect in the medial belly of the gastrocnemius, just above the myotendinous junction [35]. Patients are often unable to rise on tiptoe and show reduced strength in plantar flexion [35]. Most patients report severe pain and swelling within the first 24 hours after the traumatic event. Acute-phase physical examination frequently reveals a palpable defect in the medial belly of the gastrocnemius, located just above the myotendinous junction [34].

Although TL is common among athletes, studies show that it occurs more frequently in men over the age of 40 with poor physical conditioning and, in some cases, in work-related contexts [59]. Some authors [60] emphasize that this condition is more strongly associated with tennis-related sports activity than with occupational activity, in contrast to the so-called “tennis elbow.” Despite the characteristic clinical signs, TL may be confused with deep vein thrombosis or thrombophlebitis. Therefore, premature initiation of anticoagulant therapy may result in severe complications, such as hemorrhage, hematoma, or compartment syndrome [61,62].

Imaging techniques allow confirmation of the diagnosis, exclusion of other conditions, and assessment of the extent of the damage, all of which are essential for therapeutic management [36,63].

Injury mechanism

**Figure 4:**
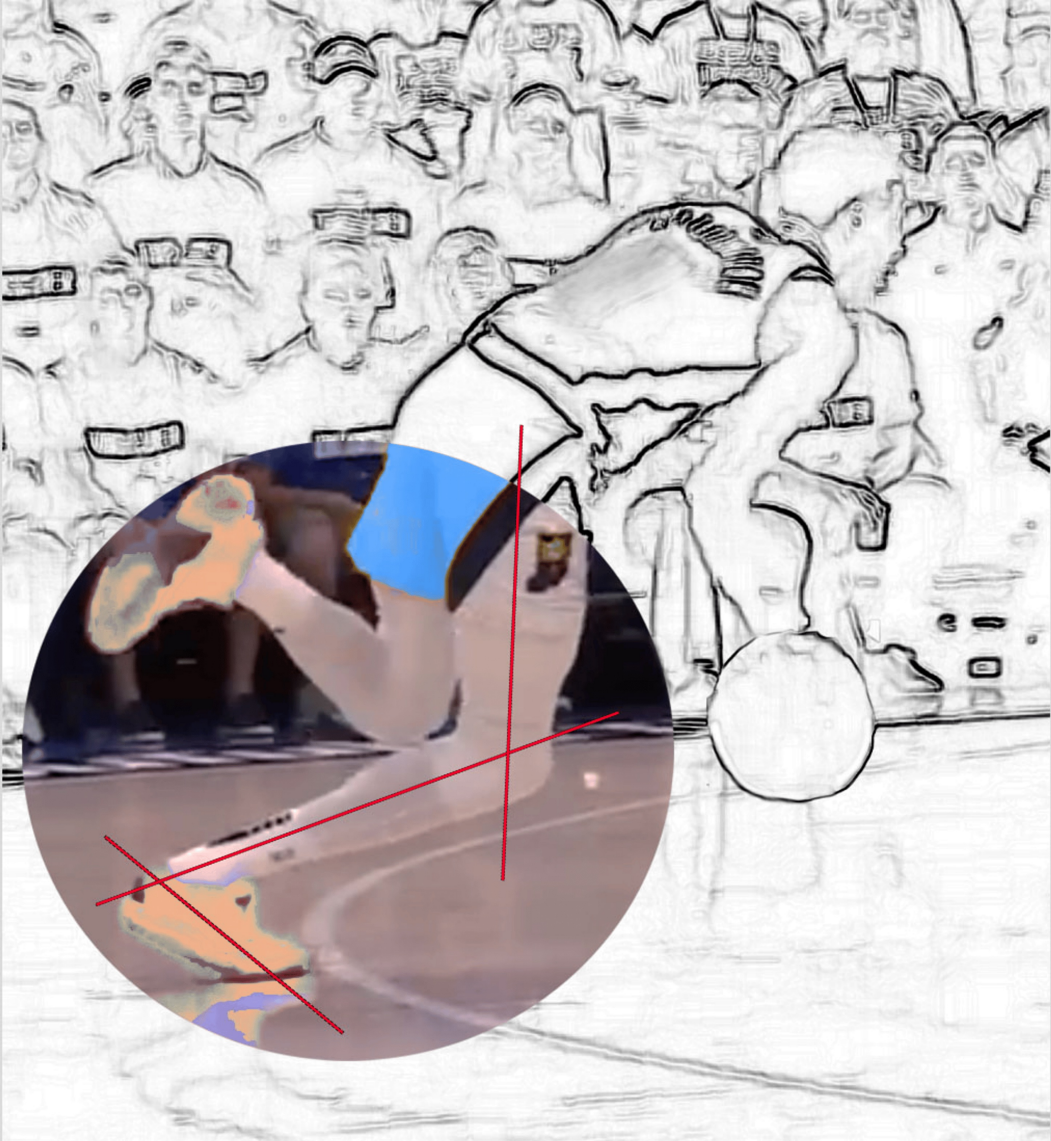
The illustration depicts the typical sports movement causing medial gastrocnemius (MG) injury A typical push-off sprinting phase during which the gastrocnemius (right limb of image) is subjected to high loads that may lead to stretching and eventual rupture. Image credit: Author Saverio Colonna. Original figure created by the author.

TL may occur after trivial movements, such as calf stretching or walking, but is more frequently caused by sudden and forceful athletic actions. It typically arises when the knee is abruptly extended with the ankle in dorsiflexion [36], a typical push-off phase in running (Figure 4) or jumping, during which the gastrocnemius is subjected to high loads that may lead to stretching and eventual rupture [64].

Functional anatomy of the triceps surae: a hypothetical etiopathogenetic key to “tennis leg”

A key question is: if the TS is a digastric functional unit, why does the lesion almost exclusively involve the medial head and not the lateral head?

To attempt an answer, it is necessary to evaluate the distribution of forces in the sagittal plane (relationship between MG and Sol) and in the coronal plane (relationship between MG and lateral gastrocnemius (LG)), an analysis that may provide insights into why the damage tends to concentrate on the medial head.

Functional Relationship Between MG and Sol

A pilot study conducted on human cadaveric material confirmed that the proximal aponeuroses of the Sol and gastrocnemius are separate structures; therefore, it was hypothesized that these two aponeuroses may undergo differential displacements, mainly influenced by knee joint position [51] (Figure 4) and by different neuromuscular activation [65].

On ultrasound images, the structure of the TS aponeurosis appears as two parallel hyperechoic lines arranged at an oblique angle to the skin surface. Reflections corresponding to the fascicles of the Sol and the MG can be observed in the classic pennate pattern, inserting into their respective aponeuroses, where the interfascicular connective tissue may be defined as hyperechoic fixed points of the aponeuroses. The longitudinal displacement of these fixed points during gradual contractions has been considered representative of the degree of deformation of the aponeurosis-free tendon complex distal to that point [50,66].

Although the AT has been studied for more than a century, interest in the structure and function of its subtendons (sAT), originating from the TS muscles, has only recently emerged [49,67-70]. The use of these studies, even if not directly related to muscle injuries occurring at the MTJ, may help us indirectly understand strain phenomena in individual units.

Clark and Franz [67] further noted that the same muscular dynamics of the TS may contribute to generating these characteristic non-uniform displacement patterns. In particular, using dual-probe ultrasound imaging, it was demonstrated that differences in shortening between MG and Sol positively correlate with differences in displacement of their respective sAT during maximal voluntary isometric contractions. However, the complex patterns of voluntary intermuscular activation may complicate the interpretation of the emerging behaviors of sAT tissues.

Through in vivo ultrasound imaging, several studies have shown non-uniform displacement patterns (i.e., greater displacements in deep tendon tissues compared with superficial ones) in the human Achilles tendon during passive ankle rotation, eccentric loading, and walking [68-70]. Based on most cadaveric studies [71-73], these findings suggest that during functional activity in young adults, the sAT of the Sol systematically undergoes greater displacement than that of the MG.

It is unlikely that MG and Sol transmit force in an equally distributed manner along their respective subtendons, making some asymmetry in levels of muscle activation necessary. Furthermore, compared with the two gastrocnemii (MG and LG), the Sol has a larger muscle volume and greater force-generating capacity, which presumably exerts a disproportionate influence on sAT displacements [74]. Thus, rigorous control of neuromuscular activation is required to better understand how the TS muscles induce differentiated behaviors within the MTJ and tendon.

The study by Lehr et al. [49], which aimed at observing localized displacements within the Achilles tendon by ultrasound, involved 10 healthy young adults with isolated electrical stimulation of MG, Sol, and both simultaneously, as well as voluntary contractions. Measurements were taken at three different ankle angles: 20° plantarflexion, 0° (neutral), and 20° dorsiflexion, with the knee flexed at approximately 20°. At 20° plantarflexion, isolated stimulation of the Sol generated a 49% greater non-uniformity of displacement between Sol and MG sAT compared with isolated MG stimulation.

For Sol stimulation, the ankle angle had a significant effect on displacement non-uniformity; specifically, peak displacement non-uniformity tended to decrease by an average of 61% from plantarflexion to dorsiflexion, probably due to increased passive tendon tension. The results confirm that sAT displacements respond consistently to specific stimulation of individual TS muscles, but these responses are strongly influenced by ankle angle. In other words, the greatest difference in mobility between subtendons (Sol > MG) is observed in plantarflexion and decreases significantly in dorsiflexion, suggesting that ankle position modulates the mechanical independence of the muscle-tendon subunits.

In plantarflexion, the tendon is more “compliant,” subtendons move more independently; in dorsiflexion, it is more “tense,” and subtendons are dragged more uniformly, with less play and more cohesion.

It should be emphasized, as previously noted, that MG muscle injuries usually occur in dorsiflexion. The behavior observed in sAT can be translated to the aponeurotic components of individual muscles. However, a direct relationship between activation patterns of individual TS muscles and the corresponding displacement patterns of specific sAT portions has not yet been clearly defined.

Understanding the degree of mechanical independence of the individual musculotendinous unit (MTU) of the TS in young, healthy subjects is fundamental for correctly interpreting negative alterations related to aging or pathological conditions. The main finding of the study by Maas et al. [75], which analyzed leg behavior in rats, confirms the hypothesis that knee and ankle joint configuration affects the magnitude of absolute displacement, relative displacement, and strain of both Sol and LG sAT.

Interestingly, the behavior of the Sol sAT was influenced not only by ankle angle variations but also by knee angle variations, despite being a monoarticular muscle. Knee extension resulted in a 28-49% reduction in Sol sAT displacement but an increase in its strain. Regardless of angular configuration, stimulation of any muscle combination generally produced displacements and strains in both LG and Sol sAT. This behavior may be explained by the connective bridges linking the two aponeurotic structures [51].

In general, Sol sAT showed greater displacement, but LG sAT exhibited greater displacements when stimulated at longer muscle lengths. However, displacements of both Sol and LG sAT decreased with knee extension independently, reaching minimum values under each stimulation condition at maximum MTU lengths. The results demonstrate that distinct Achilles sAT can move and deform differently, although they are not completely independent. Within the AT, there appears to be a delicate balance between the possibility of sliding and the mechanical connection between sAT. Since strain is a function of stress, these findings indicate that part of the force generated by the muscle fibers of one muscle is transmitted, through the inter-subtendon matrix, to the sAT of the adjacent muscle [75].

With the knee extended, the corresponding displacement of the MG aponeurosis during maximal effort significantly exceeded that of the Sol; whereas with the knee flexed, Sol displacement was significantly greater than that of the MG [51]. Interaponeurosis shear, estimated as the difference in displacement between the Sol and MG aponeuroses (Sol displacement minus MG displacement), was determined for the two knee joint positions at increasing effort levels up to the maximal common plantarflexion torque [51].

The greatest interaponeurotic shear displacement in the extended-knee position (MG displacement - Sol displacement) was 4.0 mm, while the greatest shear displacement was 2.0 mm (MG - Sol) in the flexed-knee position. These values correspond to 32% of maximum MG aponeurosis displacement in the extended-knee position and 21% of maximum Sol aponeurosis displacement in the flexed-knee position [51]. These differentiated slippages between MG and Sol aponeuroses, creating shear forces, may cause damage to the connective bridges before they fuse into the AT.

The asynchrony between MG and Sol aponeuroses may also be temporal in addition to the spatial asynchrony described above. Due to differences in contraction times [76] and shortening velocities [77] between Sol, predominantly slow, and MG, with a mixed fiber composition [78], different levels of interaponeurotic shear strain can be expected, resulting in temporal differences in contractile events, i.e., in the timing of maximal misalignment of displacement between MG and Sol muscles. Therefore, it has been hypothesized [79] that the geometric relationship between the insertion of MG and Sol fascicle aponeuroses and the differences in timing of force development between the two muscles may result in shear deformation of adjacent aponeuroses, which in turn could influence MTJ displacement. The results indicate that the magnitude of interaponeurotic shear deformation was significantly correlated with the temporal difference between the moments of maximal MG and Sol aponeurotic displacement [79].

A pilot in vitro study on five cadavers was conducted to investigate the intrinsic connective structures of the TS [51]. In all cadaveric samples, the aponeuroses of the Sol and gastrocnemius were separate structures upstream of their common junction, as the compartments were easily separable. This in vitro observation supported the initial hypothesis that sliding between Sol and gastrocnemius aponeuroses is mechanically possible during muscle contraction. However, approaching the aponeurotic junction distally, transverse collagen structures were observed, constituting a gradual increase in the stiffness of the aponeurotic connection. These transverse structures were not evident in all specimens, confirming previous observations [80,81] that there is considerable individual variability in the structure of the Sol-MG aponeurotic junction. It is likely that such structures exhibit different mechanical properties in vivo; however, the effects of cadaver fixation on the strength and stiffness of transverse collagen structures remain unclear.

Another study [79], also based on cadaveric dissections, reported that the junction of the two aponeuroses begins with thin membranes. The presence of a hematoma between the MG and Sol aponeuroses, a frequent ultrasound finding of this pathology, may indicate that rupture or damage of these interaponeurotic connections is the cause. Separation or rupture of these thin membranes could create hemorrhage between the aponeuroses, resulting in a blood collection. In other words, rupture of these interaponeurotic connections may represent a cause or underlying mechanism of hematoma formation, a phenomenon frequently detectable by ultrasound. Such damage may result from excessive tensile or sliding forces between aponeuroses, often following rapid knee extension movements, which create proximal tensile overload in the MG aponeurosis, and exaggerated foot dorsiflexion, which increases Sol aponeurosis tension in the opposite direction. Therefore, it is plausible to hypothesize that these interaponeurotic connections are involved in the process leading to TL, and their damage may explain the hematoma formation observed with ultrasound previously reported [34,82]. A more detailed investigation of the biomechanical mechanisms of these connections could provide further evidence to support this hypothesis.

Functional relationship between MG and LG

The MG and LG are often considered equivalent muscles, but neuromechanical differences between the two suggest that they may have distinct functional roles during locomotion [83]. The MG and LG appear to exhibit different neuromuscular activation patterns. In fact, during common explosive accelerations performed in sports, they show different electromyographic activations, supporting evidence that these muscles are not primarily controlled by a shared neural drive [65]. Particular attention should be paid to the earlier activation time observed in the MG compared with the LG during sprint starts, considering the higher incidence of MG muscle injuries relative to LG during explosive accelerations [65].

In Part I of this work [1], we reported in Figure 3 a schematic representation of the connective junction between muscle and tendon. In that image, the fascicles of the epimysium continue linearly with the epitenon, but this does not account for the torsions of the AT. The spiral structure of the AT has been described as a clockwise rotation (lateral) in the left limb and a counterclockwise rotation (medial) in the right limb, when observed in a proximal-to-distal direction [72,73,84]. Due to this torsion of the subtendons, the fiber arrangement in the distal region of the AT differs markedly from that in the proximal region. In the distal portion, the anterior part of the tendon is composed mainly of fibers originating laterally from the LG and/or medially from the Sol, whereas the posterolateral part is composed primarily of fibers originating medially from the MG and/or again from the Sol [72,85,86].

The torsion angle of the superficial fibers of the AT, originating from the MG, is significantly smaller than that of the deeper fibers, originating from the LG and Sol [84]. Indeed, the greatest degree of torsion of the AT is observed in its most anterior and deepest portion, composed predominantly of fibers from the LG.

**Figure 5:**
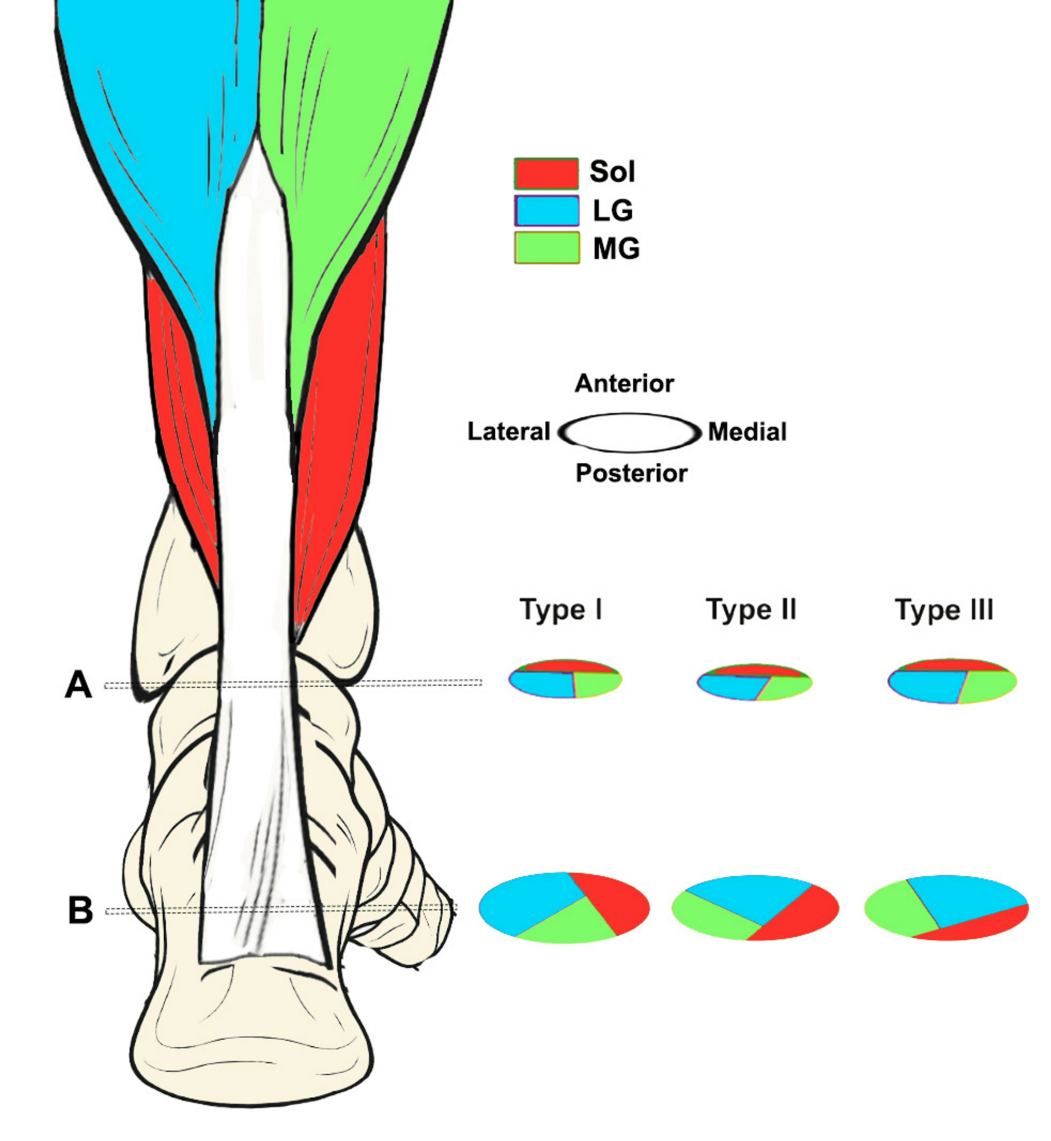
Schematic representation of the spiral structure of the Achilles tendon subtendons The image shows models of the left Achilles tendon, based on data from the study by Pekala et al. [84]. A) Section at the level of the aponeurotic junction of soleus (Sol), medial gastrocnemius (MG), lateral gastrocnemius (LG); B) upper margin of insertion into the calcaneal bone. Subtendon originating from LG (light blue); subtendon originating from MG (green); subtendon originating from Sol (red). Image credit: Author Saverio Colonna. Original figure created by the author.

As initially reported [85], three types of AT torsion have been identified: type I, with reduced torsion; type II, with moderate torsion; and type III, with marked torsion. Type I is the most common in the population, with a prevalence ranging from 50% to 84% [72,86], whereas type III is the least frequent, with a prevalence between 0% and 13% [85] (Figure 5).

Torsion affects not only the interweaving of the three subtendons with each other, but also the subunits within each subtendon [83]. Shim et al. [87] used subject-specific finite element models to simulate fascicle twist of the AT. In this study, the term fascicle was defined according to the precise nomenclature proposed by Handsfield et al. [88]: “clearly defined units in the tissue, visible to the naked eye, with diameters between 50 and 400 μm.” These are therefore micro-mesoscopic structures - i.e., primary and secondary fascicles - not the macroscopic subtendons derived from muscle bellies.

Shim et al. [87] modeled fascicular twist at different angles (0°, 15°, 30°, 45°, 60°), implementing a coordinated rotation in the material system of the finite element model. The results showed that fascicular twist: reduces localized stress concentrations by distributing them more evenly along the tendon; increases the macroscopic strength of the tendon, with rupture loads up to 40% higher than in models without twist. In summary, internal fascicle torsion is not only an anatomical concept: it has significant biomechanical and functional impact on tendon behavior. This torsion of the AT also has upstream consequences at the aponeurotic level.

The fact that the AT component connected to the MG aponeurosis undergoes less torsion than the other components could lead to a higher loading rate, with a consequent increase in impulse transmitted to the connective structures. Furthermore, greater foot supination with calcaneal varus would displace the insertion points of the lateral AT farther apart, while approximating those of the medial component. This caudal shift could be a source of asymmetric AT loading and of the corresponding digastric aponeuroses, in which the medial component would be placed under greater tension during both passive and active stretching compared with the lateral component.

Based on the torsional structure of the AT, it has been confirmed that calcaneal valgus increases strain in the medial portions of the tendon, whereas varus increases strain in the lateral portions [89].

**Figure 6:**
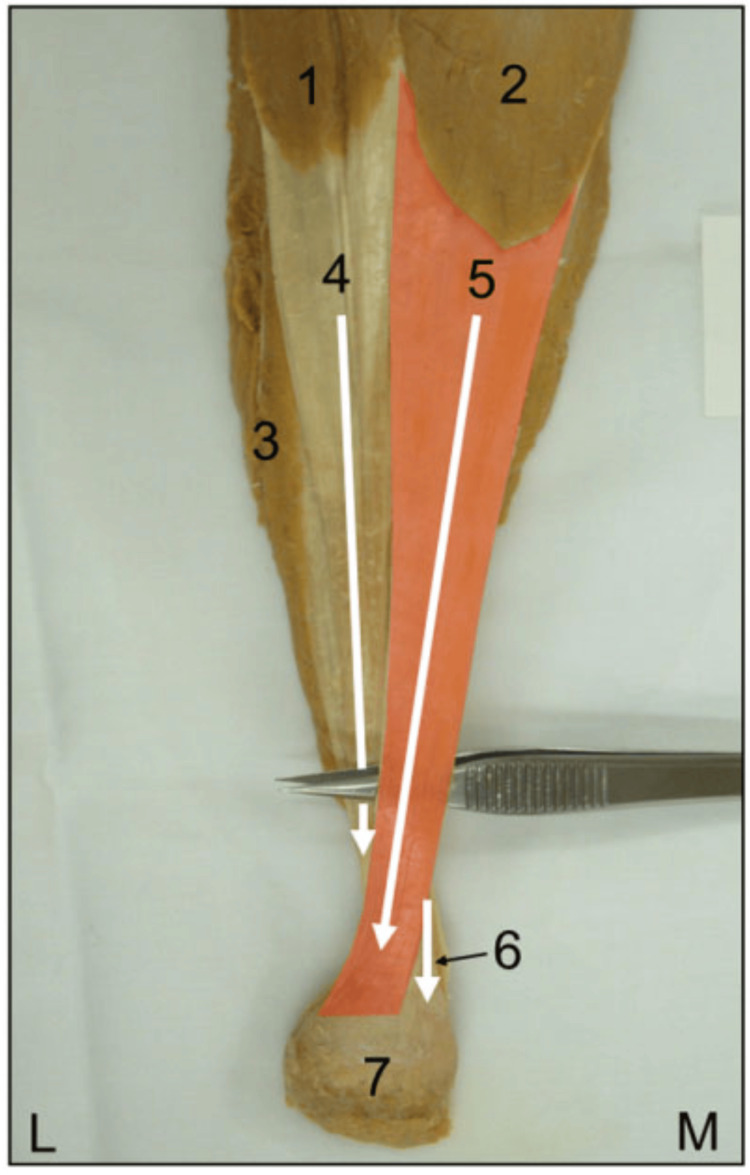
Posterior view of the left triceps surae Legend: 1) lateral gastrocnemius (LG); 2) medial gastrocnemius (MG); 3) soleus (Sol); 4) fibers from LG; 5) fibers from MG (in red); 6) fibers from Sol; 7) calcaneus; L) lateral; M) medial. Reproduced with permission from Edama et al. [86]

The presence of helical structures in the tendon appears to facilitate torsional spring-like behavior, allowing greater energy storage and subsequent release, thereby reducing peak strain, as demonstrated by smaller longitudinal deformations in the more twisted subtendons, such as LG and Sol, compared with the less twisted ones, such as MG [90]. Given that the fibers from the MG insert into the lateral portion of the calcaneus (Figure 6), calcaneal varus, often associated with foot supination, appears to be a predisposing factor for tensile overload and, consequently, for injury of the medial digastric component.

A cadaveric study [89] showed that calcaneal inversion generally increases strain in the distal-lateral tendon component and reduces it in the distal-medial component compared with the reference condition, whereas calcaneal eversion produces increased strain in the medial and reduced strain in the lateral region of the distal tendon.

Further observations [89] highlighted that, with the application of three incremental plantarflexion loads, calcaneal inversion caused a differential strain behavior between proximal and distal portions of the dorsal tendon region, with average values in the distal-lateral portion exceeding those of the corresponding proximal-lateral region.

For example, a 7.5° inversion produced mean strain equal to 250% of the proximal-lateral strain at load level 1, 200% at level 2, and 160% at level 3, whereas a 15° inversion produced mean strain equal to 550% at level 1, 165% at level 2, and 160% at level 3, confirming that foot inversion increases tension in the distal-lateral tendon component, connected to MG, compared with the proximal-lateral component, connected to LG.

During the transition from plantarflexion to dorsiflexion, strain behavior in the different regions of the AT at the calcaneal insertion shows values up to ten times higher in the posterior portion than in the anterior portion, where the anterior is predominantly composed of fascicles derived from LG and the posterior from MG [89].

Similarly, during plantarflexion, tensile load is not uniformly distributed among the components of the TS and their respective aponeuroses, since the different muscle portions have different physiological cross-sectional areas (PCSA): the Sol accounts for about 62% of the total TS PCSA, MG for 26%, and LG for 12% [74].

Since the maximal force-generating capacity of a muscle is directly proportional to its PCSA [91], it is plausible that the various components of the TS exert asymmetric loading on the AT, with the component linked to MG predominating over that of LG. However, it is still unclear what effect variations in force distribution within the TS have on AT strain distribution, or the role of tendon fascicle torsion in modulating this distribution.

Risk factors

After outlining the functional mechanisms and possible biomechanical causes of medial head injuries, it is important to consider the risk factors observed in athletes, which complement and partly reinforce this interpretative framework. It is essential to take into account the factors that predispose athletes to such injuries, as their understanding can guide both prevention and management of the condition.

In summary, several factors contribute to the risk of calf muscle injuries, as highlighted by the review of Green and Pizzari [92]. Table 2 summarizes the main determinants, ordered according to the strength of risk reported by the authors, providing a clear overview of the conditions that increase the likelihood of muscle strains in sports contexts.

**Table 2:** Risk factors for calf muscle strain injuries The table summarizes the main intrinsic and extrinsic factors contributing to calf muscle strain, ordered by their relative strength of risk based on current evidence. “Strength of Risk / Evidence” indicates the consistency and magnitude of association reported in the literature, while “Notes” provide additional context for interpretation. Data adapted from Green and Pizzardi [92].

Risk Factor	Strength of Evidence	Notes
Previous calf muscle injury	High	Most consistent predictor of recurrence
Strength imbalance between medial and lateral gastrocnemius	Moderate	Asymmetrical load on Achilles tendon and muscle fascicles
Age (older than 25–30 years)	Moderate	Increases risk of recurrent injuries
Male sex	Moderate	Higher incidence in men compared with women in high-speed sports
Limited plantarflexion / foot stiffness	Low	May predispose to overload during sprinting and change of direction
Imbalance between plantarflexor and dorsiflexor strength	Low	Influences distribution of strain in calf muscles
Excessive or rapidly increased training load	Variable	Depends on athlete conditioning and sport type
Fatigue / insufficient recovery	Variable	Predisposes to injuries during high-load sessions or congested tournaments
Reduced hamstring flexibility	Low	Associated with less adaptation of the calf to sprint stretching

A pre-existing calf injury is universally the strongest and most well-documented factor. These findings appear to be confirmed by another study conducted on athletes practicing Australian football [93], which showed that, for all muscle injuries analyzed (hamstrings, quadriceps, calf muscles), the strongest risk factor was a recent history of the same injury, followed by a previous history of the same injury.

The presence of a muscle strain in a given region also increased the risk of developing strains in other muscle groups. Age was confirmed as a risk factor for hamstring and calf injuries (even after adjustment for injury history), but not for quadriceps strains. Quadriceps injuries were significantly more common in the dominant kicking limb, whereas hamstring and calf injuries did not show differences in frequency between the dominant and non-dominant limb.

A recent article reported that calf injuries were preceded by a week with unusually high loads, associated with greater acceleration and deceleration distances and higher training volumes [94].

Use of ultrasound in the diagnosis of triceps surae injuries

Musculoskeletal ultrasound currently represents a first-line diagnostic tool in the evaluation of muscle injuries. Thanks to its ability to provide real-time information, easy accessibility, and absence of ionizing radiation, ultrasound has proven effective in assessing muscle trauma, including partial or complete.

**Figure 7:**
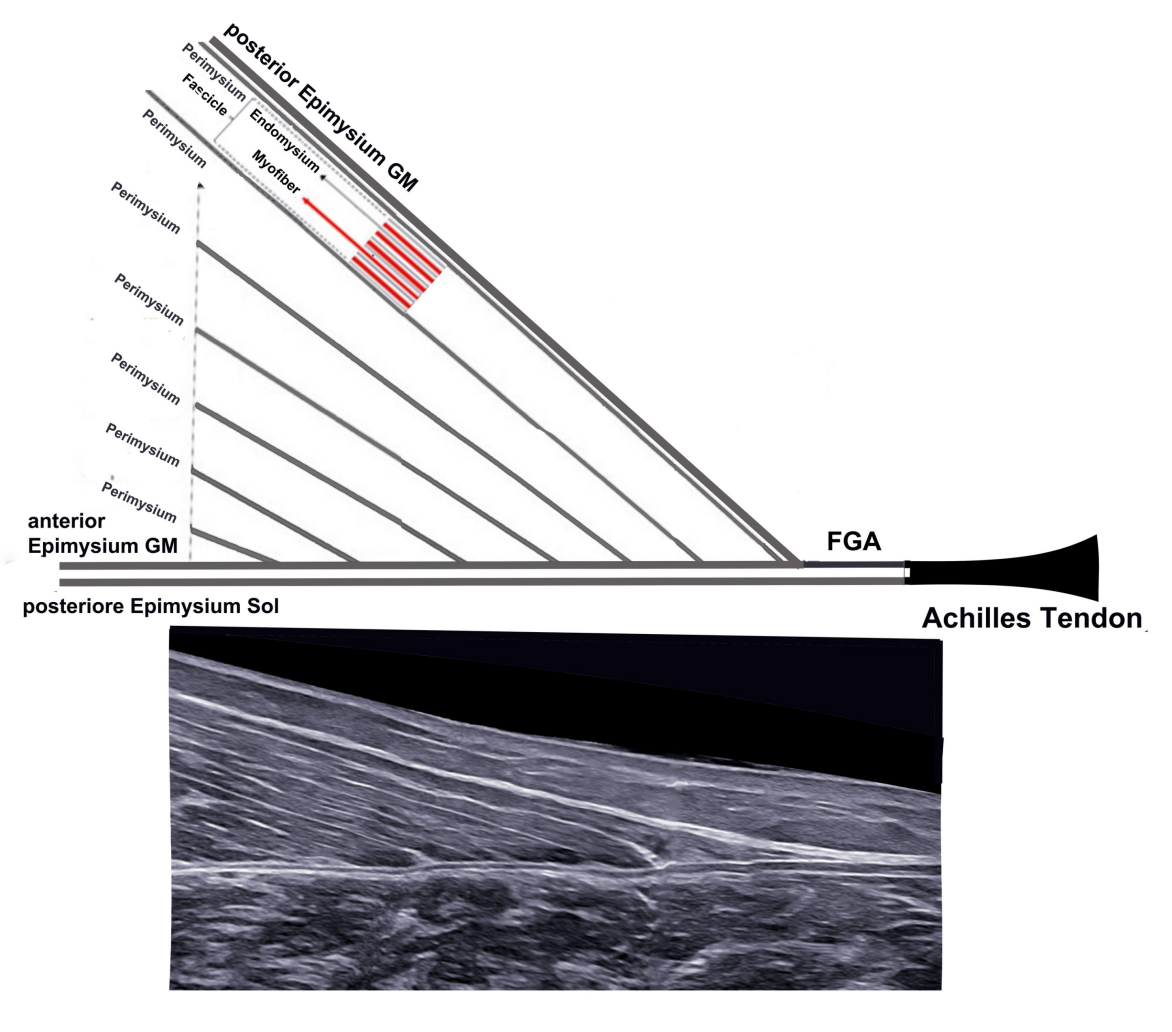
Structure and insertion of the medial gastrocnemius (MG) A) schematic representation of the connective component of the MG from the endomysium to the Achilles tendon (AT); B) B-mode ultrasound view with probe in axial position FGA = free gastrocnemius aponeurosis, GM = gastrocnemius, Sol = soleus Image credit: Author Saverio Colonna. Original figure created by the author.

In the specific case of the TS, ultrasound allows not only precise visualization of the site and extent of the injury but also discrimination of the structures involved: muscular, fascial, aponeurotic, and tendinous (Figure 7).

Rupture of the MG is sonographically characterized by disruption of the normal alternating parallel hyperechoic and hypoechoic linear pattern, typical at its insertion site. This alteration is often accompanied by poor definition of the thinned profile of the distal epimysium near its insertion on the aponeurosis [8,63].

The axial ultrasound image, which allows visualization of the entire medial belly in a single plane, has proven to be the most useful for distinguishing partial from complete injuries according to the classification of Pedret et al. [82].

Recent studies have demonstrated the high sensitivity and specificity of ultrasound in detecting partial lesions and inter-aponeurotic fluid collections, which are often difficult to identify clinically [33,95-99].

Dynamic ultrasound evaluation in calf muscle injuries also allows real-time observation of myofascial behavior under tensile load, both during contraction and during passive stretching, providing functional information not available with other methods. This approach makes it possible to identify areas of discontinuity, retraction, or separation of fibers, and to assess the degree of functional involvement of the muscle.

Furthermore, dynamic evaluation can guide recovery follow-up, tailor rehabilitation programs, and improve prediction of recurrence risk. The integration of dynamic with morphological data increases diagnostic precision and enables personalized treatment and monitoring of lesion progression over time.

Moreover, the integration with advanced techniques such as shear wave elastography allows for an additional assessment of fascial stiffness [100,101], which may be useful during the follow-up phase and in guiding the gradual return to sports activity. Several authors, however, agree that although ultrasound is very useful for muscle injuries, in some cases MRI becomes essential [102,103].

In the 141 patients evaluated by Delgado et al. [8] with clinical suspicion of TL, ultrasound revealed the following: in 94 patients (66.7%), a partial rupture of the medial head of the gastrocnemius was found, located within 2 cm of the myotendinous junction, without involvement of the plantaris tendon. Of these 94 patients, 59 subjects (62.8%) presented a fluid collection between the medial head of the gastrocnemius and the Sol muscle. In 30 patients (21.3%), ultrasound detected a fluid collection between the aponeuroses of the gastrocnemius and Sol, without sonographic signs of rupture of the TS muscle-tendon unit. In two patients (1.4%), rupture of the gracilis plantaris tendon in the middle third of the leg was observed. In one patient (0.7%), a partial rupture of the Sol muscle was found.

Deep vein thrombosis (DVT) was diagnosed in 14 patients (9.9%) as the only finding, and in seven patients (5.0%) in association with other clinical conditions. Among these seven cases, six showed a fluid collection between the aponeuroses without TS rupture, whereas in one case there was a rupture of the medial head of the gastrocnemius muscle.

From the above study, we can deduce that what are defined as strain-type muscle injuries (as opposed to direct trauma) rarely demonstrate a transverse lesion of the muscle fibers on ultrasound. Much more frequently, however, a lesion or detachment of the connective/fascial components is observed.

Classification of medial gastrocnemius injuries

The classification of MG injuries proposed by Pedret et al. [82], based on the evaluation of 115 patients, is structured around three elements: anatomical location of the injury, intermuscular hematoma, and synchronous or asynchronous MG-Sol movement.

Anatomical Location of the Lesion

Regarding anatomical location, the lesion was described in relation to the involvement of the histoarchitectural anatomy of the medial head of the gastrocnemius and divided into four types.

Myoaponeurotic injury: Myoaponeurotic injury was defined when ultrasound revealed morphological and echostructural alterations of the muscle fibers and the interposed fibro-adipose septa, corresponding to the perimysium, at the junction between the MG belly and the gastrocnemius aponeurosis (GA), without direct involvement of the GA.

**Figure 8:**
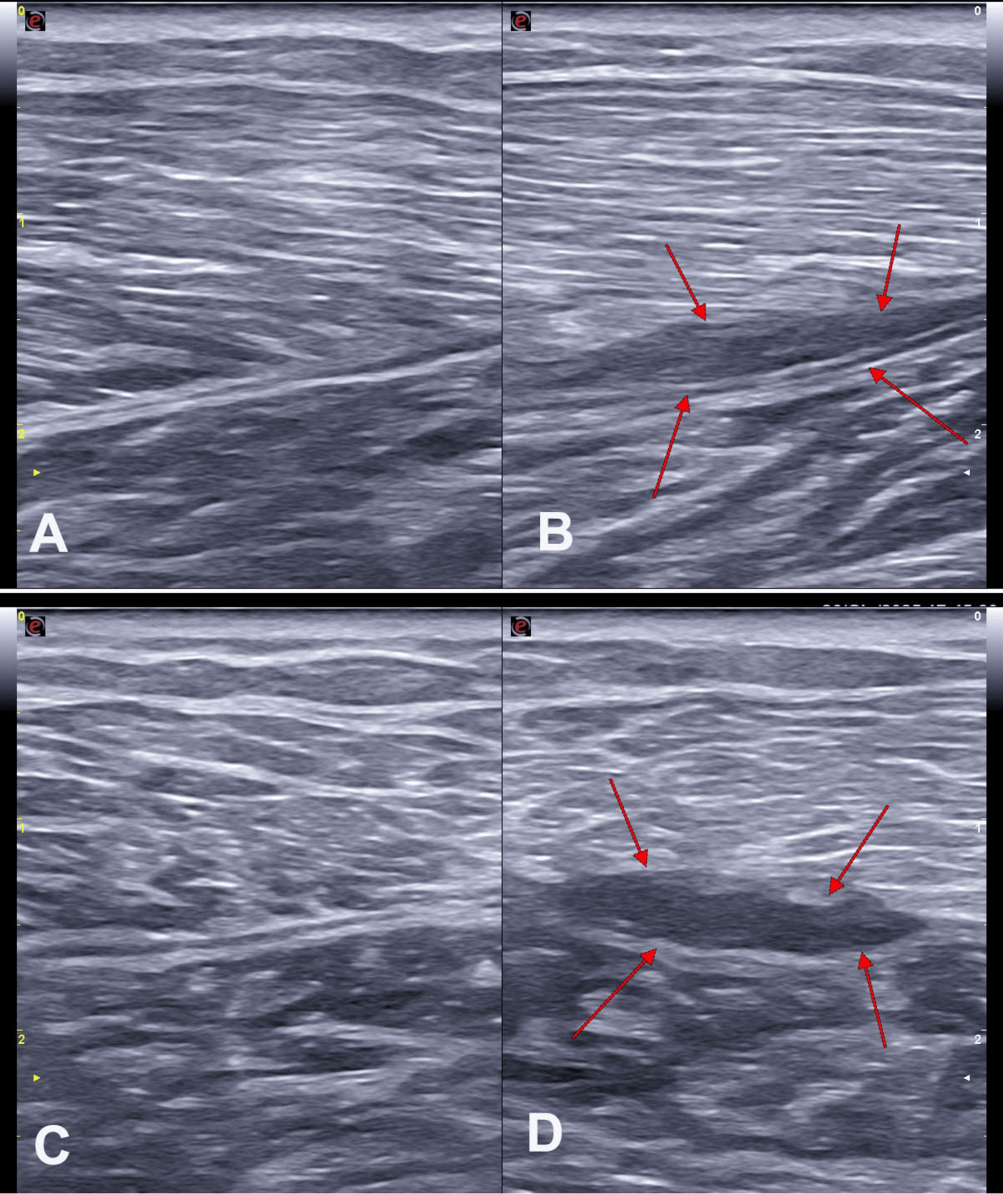
B-mode ultrasound images of the medial gastrocnemius (MG) in a subject with left MG strain A) fascial appearance of the healthy limb with probe in long axis; B) long-axis probe view of the pathological limb, with red arrows highlighting detachment of the perimysial insertion from the epimysium; C) short-axis probe view of the fascial junction in the healthy limb; D) short-axis probe view of the fascial junction in the pathological limb, with red arrows highlighting detachment of the perimysial insertion from the epimysium. Image credit: Author Saverio Colonna. Original figure created by the author.

Aponeurotic injury: Aponeurotic injury was defined when ultrasound revealed alterations in the continuity of the GA, both in short- and long-axis views, together with changes in the muscle fibers and their perimysium (i.e., myoaponeurotic lesion) (Figure 8).

The degree of aponeurotic involvement was expressed as a percentage of the total GA, specified as less than 50% (type A) or greater than 50% (type B).

Tendinous injury of the gastrocnemius aponeurosis: Tendinous injury of the gastrocnemius aponeurosis, or free gastrocnemius aponeurosis (FGA), was defined when ultrasound showed disruption in the continuity of the FGA, located immediately distal to the insertion of the MG fibers and before the formation of the proximal AT junction (gastrocnemius aponeurosis/posterior Sol aponeurosis junction).

Mixed injury: Mixed injury was defined as the combination of aponeurotic injury and tendinous injury of the FGA.

Intermuscular Hematoma

The presence of an intermuscular hematoma between the MG and Sol was also documented, and this feature appears to be the most frequent anatomo-pathological finding in TL.

Synchronous and Asynchronous Activation of the Gastrocnemius-Soleus Complex

Lesions were further classified as associated with synchronous or asynchronous movement between the gastrocnemius and Sol. During passive ankle flexion and extension, in a healthy calf the gastrocnemius and Sol move synchronously and in the same direction with proportional spatial displacement. In an injured calf, the absence or reduction of gastrocnemius movement relative to the Sol suggests a marked discontinuity of the MG-Sol complex proximal to the aponeurosis, as evidenced anatomically. This functional feature, although not yet fully studied, is highly relevant for rehabilitation, since asynchronous stretching of passive structures may lead to tensile overload predisposing one component to injury.

The results of another study [34] using ultrasound performed in the early phase after TL injury in 22 patients showed that in seven cases (31.8%) there was a partial rupture of the MG. Among these, five patients (71.4%) also presented a fluid collection between the MG and the Sol. In the remaining 15 patients, a complete rupture was diagnosed, in all cases associated with fluid collections between the two muscles. These collections showed evident hematomas, fusiform in shape and heterogeneous in appearance, located between the injured MG and the Sol aponeurosis.

The thickness of the fluid collections observed following muscle injury ranged from 4 to 16 mm (mean: 8.2 mm). In patients with complete rupture, the size of the collection was significantly greater (range: 6-16 mm; mean: 9.7 mm) than in those with partial rupture (range: 4-8 mm; mean: 6.8 mm), with a statistically significant difference (p < 0.05). At the last ultrasound follow-up after six months, among the 21 patients who initially presented with a fluid collection, 12 subjects - including the two with partial rupture associated with a fluid collection - still showed a central anechoic area (fluid) surrounded by a thick heterogeneous hyperechoic area, interpretable as reparative tissue [34].

**Figure 9:**
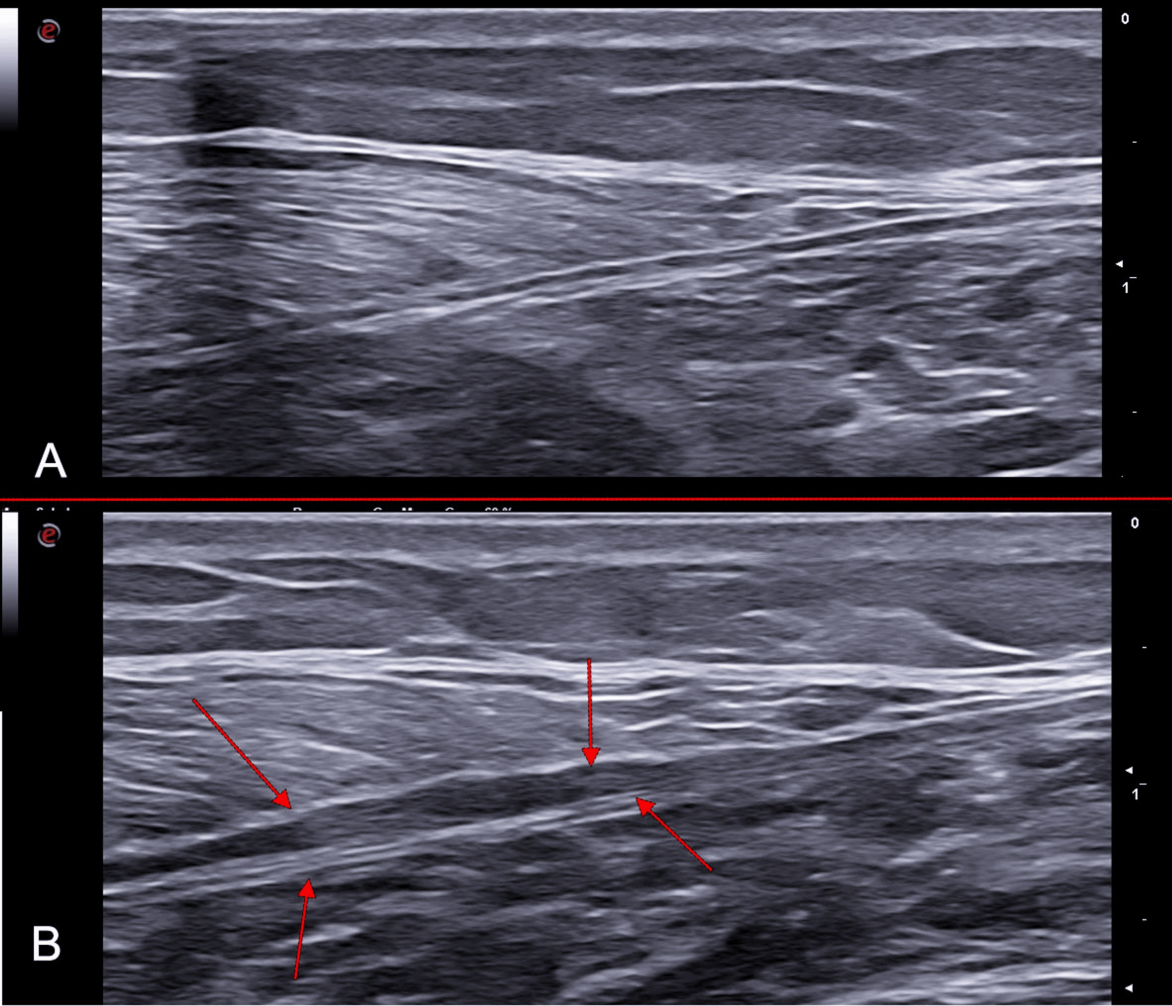
B-mode ultrasound images of the medial gastrocnemius (MG) in a subject with left MG strain A) fascial appearance of the healthy limb in long-axis view; B) long-axis view of the pathological limb. Red arrows highlight the effusion area, which may appear anechoic in the acute phase and progressively become hypoechoic during the scarring process, with detachment of the MG epimysium from the soleus (Sol) epimysium, without disruption of the epimysial fascial structures; reduced definition of the MG perimysial structures is visible in the area of greatest diastasis. Image credit: Author Saverio Colonna. Original figure created by the author.

From these findings it can be inferred that the most consistent pathognomonic sign in TL calf injuries is hemorrhagic effusion between the anterior aponeurosis of the MG and the posterior aponeurosis of the Sol. Quantification of the fluid collection after the initial trauma was defined as the maximum distance measured between the two muscles during longitudinal ultrasound scanning (Figure 9).

Primary reunion between the MG and the Sol was identified as the initial appearance of reparative tissue at the myotendinous junction between the two aponeurotic structures, observed during follow-up [34]. Several authors [104-109] agree that the greater the connective component involved in muscle injuries, the greater the severity of the lesion.

The clinical healing time was defined as the interval required for the patient to walk without pain. In addition, ultrasound examination was used to monitor over time both the variation in the fluid collection and the progression of the reparative process in cases of muscle injury.

Therapeutic indications

It is widely accepted that rehabilitation protocols for muscle injuries are designed primarily on the basis of available resources and accessible therapeutic options [110], rather than on solid scientific evidence. Clinical experience in the treatment of these injuries has shown that a wait-and-see approach, with prolonged immobilization, is not effective [2,111].

There are numerous therapeutic options for muscle injuries and, although they are widely used, many reviews have found insufficient evidence to draw definitive conclusions on any of them. Consequently, it has unfortunately become common practice to assume that what is most widely used is also what actually works [112,113].

Several physical or commercially promoted instrumental therapies are increasingly being used in the treatment and rehabilitation of conditions generically defined as muscle injuries, despite scientific evidence supporting their use being conflicting [17,111]. Although various scientific studies have attempted to propose different rehabilitation protocols, designing a specific rehabilitation pathway for each muscle injury, based on its grade and/or anatomical site, remains a complex task.

The anatomical site is particularly important because, as reported in the etiopathogenetic hypotheses, the biomechanics underlying the function of the MG are likely different from those of the Sol, which in turn differ from those of the hamstrings. In patients with muscle injuries, the diagnostic and rehabilitative approach is based on multiple factors such as age, sex, athletic demands, muscle groups involved, and type of injury. To this end, over the years several approaches have been proposed [92,114-117], all of which are based on some common criteria such as the mechanism of injury (direct or indirect) and the degree of muscle tissue involvement (structural or non-structural lesions).

Role of connective tissue and cellular responses in muscle injuries: focus on the myotendinous junction

Following a muscle injury, the body initiates a series of biological responses involving different stages of healing, each of which plays a crucial role in the process of functional recovery. This section has been included among the therapeutic indications because it is important to understand the biological timing of muscle tissue healing, with particular attention to the fascial component of the MTJ.

Phases of myofascial repair

**Figure 10:**
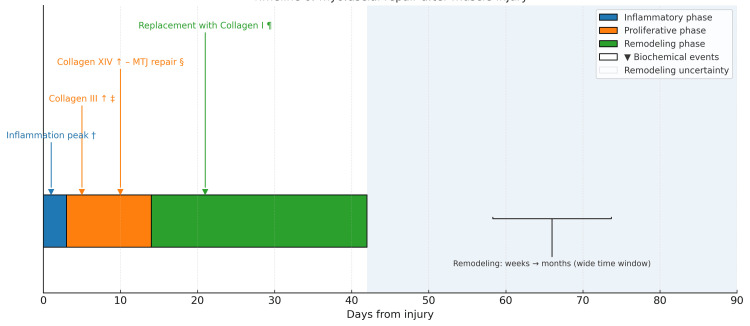
Schematic representation of the timeline of repair and biochemical events following myofascial injury. Image credit: Author Saverio Colonna. Original figure created by the author.

The three phases of muscle injury regeneration are the inflammatory/destructive phase, the healing phase, and the remodeling phase (Figure 10) [118].

Inflammatory Phase

The first stage of the healing process is inflammation, which occurs immediately after trauma and generally lasts from a few days to one week. In this phase, damaged muscle fibers release cytokines and other pro-inflammatory molecules that attract macrophages and other immune cells to the lesion site [119]. These macrophages play a fundamental role in clearing cellular debris and modulating the immune response, but also participate in the repair of the MTJ, stimulating the production of collagen and other extracellular matrix (ECM) molecules [3,116]. An increased number of macrophages in the lesion area has been documented in different muscle injury conditions, including the MTJ, where the connections between muscle fibers and connective tissue are crucial for muscle functionality [7].

Proliferative Phase

The second stage, corresponding to the proliferative phase, is characterized by regeneration and synthesis of new muscle fibers as well as repair of damaged connective structures. Collagen plays a decisive role in creating a support network for new muscle growth. Increased production of collagen XIV, especially in the endomysium, has been observed to strengthen the MTJ and facilitate the transmission of mechanical force across muscle and tendon [120,121]. This reinforcement mechanism is important for preventing future injuries, since connective structures not only protect muscle fibers but also actively contribute to the cycle of lateral force transmission.

Remodeling Phase

The remodeling phase, which extends over time, is the stage in which the ECM and muscle fibers adapt to more intense mechanical loads. In this phase, the increase in collagen in the MTJ region and its interaction with other proteins such as tenascin-X allows for greater mechanical resistance and improved tension distribution [122]. Although collagen XIV may strengthen tissue structures, some evidence suggests that this increase may be transient, reflecting a remodeling process that prepares tissues for optimal functional recovery [7,120].

An increase in macrophages within a few days of intense exercise or injury has been reported in human skeletal muscle when sampling was performed in the central muscle region using needle biopsy [123,124], in the perimysium and endomysium, with clear evidence that these cells specifically adhere to the MTJ of the hamstrings [7]. This finding is consistent with the role of macrophages in repairing the MTJ region both at rest and in response to mechanical load. After four weeks of high-intensity resistance exercise, a greater amount of collagen XIV was observed in the endomysium of the hamstrings compared to controls, suggesting that this type of collagen may play an important role in reinforcing the tissue junction.

Collagen XIV, as well as collagen XII, possesses domains capable of connecting ECM structures such as collagen fibrils and tenascin-X [125]. For this reason, collagen XIV can be considered a sort of “small anchorage” linking larger structures together, forming wide ECM enclosures.

Several studies have indicated that the endomysium plays a relevant role in force transmission through a lateral pathway [126]. In this mechanism, much of the force is transmitted as shear stress, thereby reducing overall stress on the ECM and MTJ.

It is possible that an increase in collagen XIV may reinforce the ECM scaffold near the MTJ and potentially optimize the lateral force transmission pathway, thus reducing the load on the MTJ and protecting this structure from strain injuries. Alternatively, the collagen XIV increase induced by training may be transient and reflect an ongoing remodeling process of other matrix proteins.

Muscle or fascial lesion and return to play (RTP)

While the vulnerability of extramuscular connective tissue during athletic activity has been convincingly demonstrated, it is not yet clear whether fascial injuries result in different RTP timelines. The studies included in a systematic review [127] that investigated the association between fascial damage and athlete downtime produced heterogeneous results without conclusive evidence, partly due to the heterogeneity of statistical procedures and analytical aims across individual studies, which did not allow for meta-analytic pooling.

In a study focused on the extent of fascial injury, Werner et al. [128] compared lesion size in athletes with short RTP (less than two weeks) versus long RTP (more than two weeks). On average, lesions were three times larger in the latter group, with a correspondingly longer period of inactivity. Other authors [129-131] reported similar results, in which patients with larger fascial lesions required longer RTP timelines (Table 3). In a sample of 100 patients with 114 lesions, connective tissue involvement was observed in 63/100 cases. In those with complete connective tissue disruption (grade 3), the mean RTP was 48 days, compared with 25 days for grade 2 lesions, 17 days for grade 1, and eight days for grade 0 [130].

**Table 3:** Rehabilitation modalities and reported recovery timelines Legend: ROM = range of motion; RTP = return-to-play. This table summarizes rehabilitation modalities with indicative timelines derived from [129–131], highlighting the need for criterion-based progression rather than fixed time points.

Rehabilitation modality	Key features	Reported recovery outcomes (RTP days)
Stretching & mobility	Early controlled stretching, gradual ROM restoration	RTP ~ 20–25 days in mild/moderate cases
Eccentric strengthening	Progressive loading of triceps surae, emphasis on aponeurotic compliance	RTP ~ 25–35 days with lower reinjury rates
High-intensity resistance & functional loading	Maintained ≥8–12 weeks; load progression linked to tendon adaptation	RTP ~ 30–40 days; improved long-term resilience
Criterion-based RTP testing	Functional assessment before return (strength, endurance, no pain)	Safer RTP decision-making; reduced recurrence risk

The authors concluded that the proportion of connective tissue damage relative to intact tissue may be used to estimate and guide RTP timelines in calf muscle injuries.

Another study [129] compared RTP between muscle injuries and connective tissue injuries (fascia and intramuscular connective tissue, including tendon). Reported data showed that athlete downtime was significantly longer in cases of connective tissue damage (7.6 weeks) compared with muscle damage alone (3.9 weeks).

Other studies [108,132] reached partly different conclusions, reporting no significant differences between lesion types, although fascial lesions tended to show longer recovery times. Overall, the literature suggests a trend toward longer RTP in the presence of fascial injury, but further studies are required to confirm this observation [127].

Rehabilitation protocols

While a large body of literature exists on the prevention and treatment of Achilles tendinopathy and hamstring injuries through therapeutic exercise [17,133-139], the evidence for calf muscle injuries is much more limited [17,140,141]. For this reason, this section frequently refers to research conducted on hamstring strain and Achilles tendon pathology, acknowledging that the approaches are not entirely interchangeable, as tendon and aponeurotic deformation may differ [142,143], and the effects of training may vary between these structures.

Although the effect of resistance training on the tendon-aponeurosis complex in young adults has been investigated [144,145], information regarding modulation of the structural stiffness of the myofascia remains scarce. However, changes in the magnitude and rate of active force production induced by training may be influenced by the behavior of the series elastic structures, particularly the free tendon. Therefore, to assess the impact of resistance training on the performance of the muscle-tendon unit as a whole, it is necessary to consider the properties of tendon structures and integrate them with ultrasound analysis of muscle architecture.

Current rehabilitation paradigms for muscle strain in general include various methods, particularly eccentric training and neuromotor control exercises [16]. Even if future studies do not confirm prolonged downtime due to fascial injuries, targeted interventions addressing the tissues involved in different subgroups (purely muscular lesions versus predominantly collagenous lesions) may still promote faster recovery.

Therapeutic exercise programs can be organized according to three main criteria: movement modality, load intensity and duration, and joint angle at which they are performed.

Movement modalities

Since the first work by Carwin and Stanish in 1984 [146] on the use of eccentric exercise in tendinopathy, numerous subsequent studies have further investigated the topic, with the most notable being that of Alfredson et al. [147], which explored eccentric calf exercise for Achilles tendinopathy.

The movement modalities used in training and therapeutic exercise programs are four: isometric, concentric, eccentric, and plyometric. Each of these modalities has specific characteristics that make it useful in particular phases of rehabilitation. Table 4 summarizes the main features of each of the four modalities. Data adapted from Franchi et al. [148], Hody et al. [149], Booth and Orr [150], and Seiberl et al. [151].

**Table 4:** Comparative table of muscle contraction modalities Definition, Biomechanical Characteristics, Clinical Example and Physiological Adaptations of the main exercise modalities

Modality	Definition	Biomechanical Characteristics	Clinical Example	Physiological Adaptations
Isometric	Tension is developed without any change in muscle length.	Force = resistance; no joint movement.	Plank, static hold in post-surgical rehabilitation.	Maintenance of muscle trophism, joint stabilization.
Concentric	The muscle shortens while overcoming resistance.	Force > resistance; shortening of muscle fibers.	Lifting phase in a biceps curl.	Increased dynamic strength, hypertrophy.
Eccentric	The muscle lengthens under tension, controlling the movement.	Resistance > force; controlled lengthening of fibers.	Descent phase in a squat, Alfredson protocol.	Tendon strengthening, injury prevention, improved motor control.
Plyometric	Rapid stretch–shortening cycle followed by an explosive phase.	Eccentric contraction immediately followed by concentric contraction; utilizes stored elastic energy.	Depth jump with immediate upward push.	Increased power, reactivity, and neuromuscular coordination.
Auxotonic	The muscle changes length while the tension it develops also changes throughout the movement.	Combination of isotonic (length change) and variable resistance due to elastic or mechanical load.	Resistance band exercises, free-weight lifts with variable load curves.	Strength gain across full range of motion, improved coordination under variable resistance.

Isometric modality

Since isometric contractions allow for precise and controlled application of force within pain-free joint angles [152], isometric training (IT) is often employed in clinical contexts. It is therefore essential to understand whether such stimuli generate relevant adaptations at the muscle-tendon level. Systematic studies and reviews indicate that IT, particularly when performed under tensioned muscle conditions, promotes increases in tendon stiffness and elastic modulus, as well as a certain degree of muscle hypertrophy, thereby contributing to greater resistance of the muscle-tendon complex [152]. IT has also been shown to be effective in enhancing maximum joint angle-specific strength, with good transferability to dynamic tasks such as running, jumping, or sprinting [153].

In rehabilitation, IT allows for selective and gradual application of load, favoring tendon adaptation without subjecting the injured area to excessive stress [154].

Concentric modality

In myofascial injuries, concentric contraction plays a strategic role particularly in the early phases of rehabilitation, when it is necessary to apply force while reducing tension on the injured fascial component, although this always depends on load.

The muscle shortening characteristic of concentric contraction limits strain on the myotendinous junction and on intramuscular connective laminae compared with eccentric and plyometric contractions, allowing safe stimulation of contractile capacity [152]. Since isometric contractions allow for controlled application of force within pain-free joint angles [152], isometric training (IT) is frequently employed in clinical practice.

At the adaptive level, concentric work can significantly increase pennation angle, thereby optimizing the muscle’s ability to generate force [155]. This adaptation, relative to fascicle length, is particularly predominant in concentric modalities and is supported by experimental evidence in both young and older subjects [156].

The increase in pennation angle promotes an augmentation of physiological cross-sectional area (PCSA) without excessively stressing the injured connective component during the initial phase of rehabilitation.

Relevance of eccentric vs plyometric loading in tendinopathy and muscle injuries

Eccentric exercise has represented, since the first work by Carwin and Stanish in 1984 [146], the therapeutic paradigm in tendinopathy for nearly four decades, particularly due to the protocol proposed by Alfredson for Achilles tendinopathy [147]. Subsequent studies [157] have confirmed the effectiveness of protocols based on eccentric loading, although systematic reviews and meta-analyses have shown that isolated eccentric loading is not always superior to combined strengthening programs or heavy slow resistance (HSR) training in tendon injuries [158].

Eccentric contraction is the modality that generates the greatest strain on both muscle fibers and fascial networks, and must therefore be introduced with caution in myofascial injuries, preferably after an initial isometric and concentric phase [148,159].

In the context of muscle injuries, eccentric training has been hypothesized to improve the muscle’s ability to tolerate active lengthening and reduce the risk of recurrence [159]. Under controlled conditions, eccentric contractions stimulate increases in fascicle length and the addition of sarcomeres in series, thereby enhancing tolerance to the muscle lengths at which injuries typically occur [160,161].

After 18 eccentric exercise sessions over a seven-week period, muscle thickness and fascicle angle of the MG increased significantly at rest and during contraction, whereas fascicle length increased only at rest, with no variation during contraction. Tendon stiffness of the gastrocnemius also increased significantly. These findings suggest that muscle architecture and tendon mechanical properties respond differently to strength training [140].

However, this approach focuses mainly on the muscle as a contractile element, while often neglecting the specificity of sports movements, which typically occur under high-velocity SSC conditions [151]. The SSC is characterized by an eccentric phase immediately followed by a concentric phase, exploiting the recovery of elastic energy stored in the connective components of the MTU, particularly the series elastic components (tendon and aponeurosis), with contributions also from parallel elastic components and titin [151,162].

Plyometric training, which is based on the SSC, induces adaptations in tendon stiffness and neuromuscular coordination specific to explosive activities, making it more appropriate than slow eccentric loading in contexts where the actual demand is dynamic and reactive [144,163].

From an epidemiological and biomechanical standpoint, acute muscle injuries in sprinting, jumping, and change-of-direction sports typically occur under conditions of rapid active lengthening, high velocities, and submaximal but explosive loads, such as during the terminal swing phase of sprinting in the posterior thigh muscles [164-166].

In these scenarios, the loading pattern more closely resembles high-intensity plyometric exercise rather than controlled eccentric contraction. Moreover, imaging evidence and histopathological studies show that many of these injuries predominantly involve connective tissue (MTJ, intramuscular connective tissue, and fascia) rather than muscle fibers per se [127,167,168].

In conclusion, joint stiffness and active muscle stiffness increased after plyometric training but not after isometric training. Furthermore, tendon extensibility during ballistic contractions improved following plyometric training, whereas tendon stiffness increased after isometric training. These results suggest that the changes in muscle and tendon properties induced by plyometric training favor performance in exercises involving the stretch-shortening cycle.

In the study by Kubo et al. [163], eleven subjects completed an ankle plantarflexion training program three times per week for 12 weeks, with one limb performing plyometric training and the other isometric training. Subjects were evaluated before and after training using ultrasound, dynamometry, and force platforms. The results indicated that plyometric but not isometric training increased tendon extensibility during ballistic contractions and active muscle stiffness during rapid stretches, and that these modifications may be linked to improved performance in SSC-based exercises [163].

This finding further supports the rationale for a rehabilitative and preventive approach that incorporates stimuli aimed at enhancing the resilience of connective components, such as those induced by plyometric training, even though some authors have not observed a specific benefit [169]. It is important to note that the meta-analysis by Bohm et al. [169] referred specifically to tendon adaptation and may not directly apply to other connective structures such as aponeuroses and intramuscular fascia, which exhibit different mechanical properties and adaptation times. The fact that plyometric training is not optimal for tendon adaptation may be related to the intermittent, high-velocity nature of the stimulus, which provides less “time under tension” compared with prolonged high-load contractions. However, in explosive-type muscle injuries, where the structures primarily involved are often the connective components of the MTJ or fascia, progressive plyometric stimulation may be more specific to the objective of restoring function and mechanical resilience under conditions similar to those that produced the injury.

Because plyometric training closely reproduces the mechanisms underlying muscle injury, its implementation requires particular caution. Two main strategies have been proposed. The first is a traditional gradual progression, beginning with isometric contractions, then introducing concentric-isotonic movements, followed by eccentric loading, and only in the final stage incorporating high-intensity plyometric drills once pain, strength, and neuromuscular control allow safe execution. Continuing with progressively heavier but slow-loading schemes does not appear to be effective for restoring the function of connective tissues such as tendons [170]. The second strategy involves an early but low-intensity introduction, using attenuated forms of plyometric loading that reduce body weight or employ devices to control excursion and minimize load (e.g., seated calf raises or leg-press variations with light loads). This approach aims to stimulate the elastic components at an earlier stage without placing excessive stress on the healing tissue.

Load intensity and duration

A systematic review and meta-analysis [169] investigating the chronic effects of mechanical loading on human tendon adaptation in vivo included 27 studies with a total of 37 distinct exercise interventions. The findings demonstrated that tendons are highly sensitive to increased mechanical load and adapt primarily through modifications of their material properties rather than morphological changes. High-intensity loading (i.e., elevated levels of muscle contraction force) was most effective in promoting tendon adaptation, and intervention durations longer than 12 weeks yielded greater benefits compared to shorter protocols. The type of muscle contraction, however, did not significantly influence the outcome.

The same review by Bohm et al. [169] emphasized that tendons respond particularly through changes in their material properties rather than in cross-sectional area. High-intensity loads and durations of at least 12 weeks were the most effective stimuli for these adaptations. More recent studies suggest that significant changes in tendon stiffness may occur after only 8 weeks of adequate stimulation, with material properties playing a predominant role [171]. An updated meta-analysis further confirmed that increases in elastic modulus were much more pronounced than changes in tendon cross-sectional area, underlining the importance of material adaptations in tendon stiffness [172].

From these data, several practical considerations can be drawn. First, the minimum effective dose appears to require at least 8-12 weeks of continuous stimulation, as shorter interventions are unlikely to induce substantial structural adaptations. Second, load progression should be gradual but consistent, avoiding prolonged periods of low-load training that seem insufficient for tendon and connective tissue adaptation. Third, specificity to the target structure must be taken into account: while tendon adaptation requires heavy loading and relatively long time under tension, other connective structures such as fascia and aponeuroses may respond to shorter, reactive stimuli such as plyometrics, provided they are introduced in a progressive manner. Finally, load prescription should integrate multiple variables, including weekly frequency, time under tension, and joint angle specificity, which together influence adaptive responses and must be modulated according to the stage of recovery and the injured structure.

These considerations indicate that, in the rehabilitation of muscle and tendon injuries, it is not sufficient to determine which type of contraction to employ; rather, careful calibration of load magnitude, duration, and progression is required to maximize structural and functional adaptations.

Joint angle and fascial tension in different contraction modalities

Joint position plays a crucial role in fascial tension and recurrence risk. In the case of MG injury, damage typically occurs with the knee in extension and the ankle in dorsiflexion, which corresponds to the condition of maximal fascial tension. A study by Liu et al. [173] demonstrated that dorsiflexion induces non-uniform tensile stress across muscles, tendons, and the plantar fascia, thereby supporting the rationale for initially applying loads in plantarflexion to reduce fascial strain and promote safe adaptation.

From a rehabilitative progression perspective, the recommended sequence for managing joint angle in MG myofascial injuries is presented in Table 5.

**Table 5:** Rehabilitation progression Fascial loading according to joint angle and type of muscle contraction, from plantarflexion to dorsiflexion MTU = musculotendinous unit

Phase	Joint Position	Rationale
1	Plantarflexion	Reduces fascial tension and provides safer initial loading conditions.
2	Neutral (0°)	Intermediate step allowing progressive adaptation.
3	Dorsiflexion	Highest fascial strain; introduced only after sufficient adaptation of the MTU.

This order - from plantarflexion toward dorsiflexion - may enhance the safety of rehabilitation, as it reduces fascial loading during the initial phases and allows for more robust structural adaptation along the MTU.

Usefulness of stretching in fascial muscle strains

For connective tissue, in addition to strengthening exercises, it may be useful to include static or dynamic stretching in the program [174-176]. Several indexed studies support the inclusion of stretching in rehabilitation protocols for muscle strain injuries. In a randomized clinical trial, Kim et al. [177] demonstrated that, in patients with hamstring injuries, a rehabilitation program incorporating static and active stretching, range of motion exercises, and strengthening led to a significant reduction in pain, as well as improvements in flexibility and isometric strength. Moreover, a review of rehabilitative practices [176] highlighted that static stretching is more effective than dynamic stretching in the treatment of strain injuries, suggesting that a more intensive stretching approach may accelerate recovery.

Edama et al. [86] studied the macroscopic anatomy of the triceps surae in Japanese cadavers and verified, through ultrasound on volunteers, a stretching protocol specifically targeting the MG: positioning the limb with the knee extended and the foot/ankle in dorsiflexion plus inversion resulted in a selective and efficient elongation of the MG. This approach produced a significant reduction in pennation angle and an increase in muscle fiber length, suggesting therapeutic applicability for injury prevention and optimization of rehabilitation treatments, as well as for preventing future MG injuries [86].

It should be noted, however, that when attempting to identify the optimal ankle position to selectively stretch the myofascial structures of the MG, one should avoid the error of excessively seeking and maintaining the dorsiflexion + inversion posture. As highlighted in the biomechanics chapter [89], and as will be discussed again in the section on manual therapy, this position is crucial in the pathomechanics of MG strains. Therefore, reinforcing this foot/ankle posture should be avoided in the early phases of rehabilitation.

Regarding the effectiveness of stretching, different findings were reported in another study [178], where static stretching performed for six weeks at different ankle joint angles did not significantly modify either strength or MG architecture (thickness and pennation), as evaluated by ultrasound.

Although no published studies have specifically investigated hamstring stiffness as a direct risk factor for medial gastrocnemius strain (“tennis leg”), some anatomical and functional considerations support a plausible relationship. Anatomical studies have demonstrated fascial continuity between the hamstrings and the triceps surae. Cadaveric dissections have shown that the semitendinosus presents an accessory fascial band extending distally toward the crural fascia, in close continuity with the connective layers enveloping the medial gastrocnemius. In a study of 23 limbs, such a connection was identified in 22 cases, with a mean width of approximately 2.6 cm and located about 7 cm from the tibial insertion of the semitendinosus [179]. Other studies [180,181] have confirmed that the fascial expansions of the medial hamstrings (semitendinosus and gracilis) merge with the crural fascia, which in turn is closely connected with the epimysial fasciae of the triceps surae compartment [179,182]. These observations provide an anatomical substrate for the transmission of tension between the hamstrings and the medial gastrocnemius: the semitendinosus tendon shows continuity with the crural fascia and, through its aponeurotic expansions, with the epimysial fascia of the medial gastrocnemius, suggesting that increased hamstring tension may transmit mechanical stress distally [182-184].

This functional connection has also been demonstrated in an in vivo study (ultrasound + motion tracking) that measured a significant correlation between pelvic rotation and displacement of the deep fascia of the medial gastrocnemius. Greater hamstring elasticity was associated with reduced fascial displacement of the MG, suggesting a functional link along the posterior fascial chain [185]. Functionally, the posterior myofascial chain has been widely described as a continuous kinetic and tensional system linking the hamstrings, gastrocnemius-soleus complex, and plantar fascia, supporting the hypothesis that stiffness in one segment may influence load distribution along the entire chain [4,186-188].

From this perspective, if a functional - and therefore also dysfunctional - relationship exists between hamstrings and plantar fascia, as several studies have already demonstrated [189-191], it seems difficult to exclude a connection with the triceps surae, which is located anatomically and functionally between the two. Therefore, it is reasonable to hypothesize that hamstring stiffness may contribute to altered load transmission along the posterior chain, predisposing the gastrocnemius to overload during eccentric contractions in knee extension and ankle dorsiflexion, the typical mechanism of tennis leg [8,192]. Based on these considerations, stretching the hamstrings-either through self-stretching exercises or manual fascial lengthening techniques-may be beneficial.

Manual therapy

Manual therapy for tennis leg may target the fascial component, complementing the elongation achieved through self-managed stretching, or focus on the articular component. For the fascial component, some studies [193] have reported favorable results of fascial manual therapy in increasing hamstring extensibility.

Articular mobilization techniques may also be useful in managing certain tendinopathies of the human body [194-196]. Since motion restriction is a possible risk factor for Achilles tendinopathy [197], it appears reasonable to include mobilizations and joint manipulations in the conservative management of Achilles tendinopathy and, by extension, in myofascial injuries of the MG. Altered mobility of the ankle and its joints can contribute to the development or persistence of Achilles tendinopathy [198,199].

Some runners with Achilles tendinopathy, when treated in physiotherapy, were found to have hypomobility of the hindfoot. When joint mobilization and manipulation techniques were added to eccentric exercise programs, patients experienced immediate improvements in symptoms and function, maintained both at discharge (12 weeks) and at nine-month follow-up. Manual therapy thus appears to be a safe and effective intervention in the rehabilitation of chronic tendinopathies [197].

**Figure 11:**
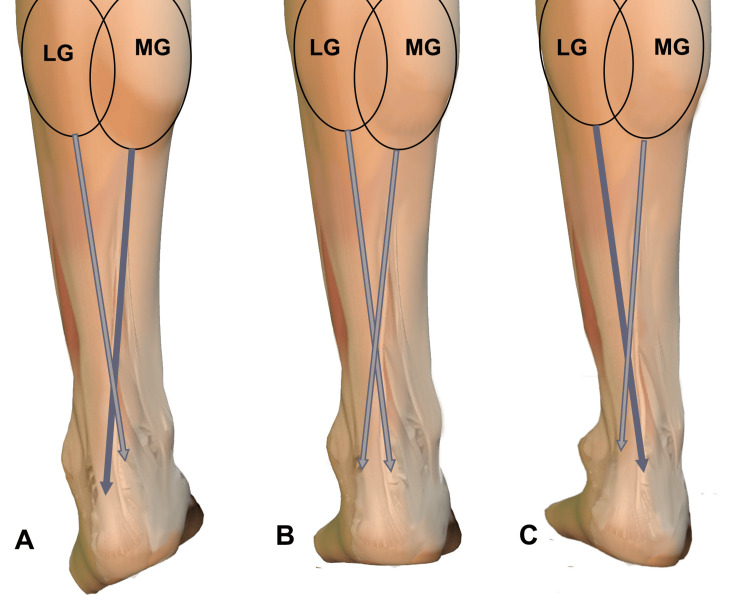
Schematic representation of fascial tension vectors of the medial gastrocnemius (MG) and lateral gastrocnemius (LG) according to changes in hindfoot alignment A) varus/inversion axis – increased tension of the MG aponeuroses; B) neutral condition – balanced tension of the MG and LG aponeuroses; C) valgus/eversion axis – increased tension of the LG aponeuroses. Image credit: Author Saverio Colonna. Original figure created by the author.

The literature reports that patients with chronic lateral ankle instability secondary to sprains frequently exhibit increased hindfoot varus [200,201]. For this reason, in patients with tennis leg it is advisable to assess the talocrural and especially the subtalar joints. If marked rigidity in varus/supination is observed - a position that increases tensile stress on the MG [89] (Figure 11) - manual treatment and/or specific exercises aimed at correcting calcaneal alignment are indicated. Following this biomechanical logic, load progression during therapeutic exercise programs should begin with the foot in pronation/valgus and later, especially in the final stages, in supination/varus.

Along the same lines, regarding hip positioning during standing heel raises with a barbell, internal rotation preferentially activates the MG, while external rotation preferentially activates the LG [202].

Nutritional support for connective tissue in myofascial injuries

Recent studies suggest that oral supplementation with type I hydrolyzed collagen, in combination with substances such as arginine, MSM, and bromelain, may promote regenerative processes at the myotendinous junction and within fascial tissues [203]. Daily intake of collagen peptides, even at moderate doses (about 15 g/day), has been associated with improvements in collagen synthesis and joint function, although without significant effects on overall muscle protein synthesis [204]. In post-exercise contexts, short cycles of collagen supplementation have been shown to accelerate functional recovery and reduce muscle soreness, supporting the paradigm of faster and less painful fascial remodeling [205]. Moreover, micronutrients essential for collagen synthesis-such as vitamin C, vitamin D, zinc, and copper-have been identified as critical for effective tissue repair in musculoskeletal settings [206].

## Conclusions

The growing recognition of the fascial system’s role in muscle injuries underscores the need for an integrated diagnostic and therapeutic approach. Distinguishing between muscle fiber and fascial involvement is not a purely semantic exercise but a crucial clinical step, as connective tissue damage has distinct implications for prognosis, rehabilitation strategies, and return-to-play timelines. Advanced ultrasound, supported by updated classifications and anatomo-functional knowledge, is now a cornerstone for diagnosis and follow-up, allowing clinicians to identify connective versus contractile lesions and adapt treatment accordingly.

Future research should clarify the biomechanical mechanisms underlying fascial involvement, develop rehabilitation protocols tailored to the tissues most affected, and refine preventive strategies based on biomechanics and individualized training. A comprehensive approach that integrates imaging, exercise therapy, manual interventions, and biological healing timelines may reduce recurrence, optimize recovery, and enhance long-term athletic performance.

In conclusion, this review highlights the pivotal role of connective tissue structures in the pathophysiology of tennis leg, shifting the focus beyond a purely muscle-centered perspective. Integrating histological, biomechanical, and imaging evidence provides a more accurate diagnostic framework and supports criterion-based rehabilitation strategies that emphasize progressive loading and aponeurotic involvement. These insights have direct clinical implications, offering clinicians tools to improve diagnostic accuracy, guide tailored rehabilitation programs, and ultimately optimize patient outcomes and return-to-play decisions.
